# Trazodone rescues dysregulated synaptic and mitochondrial nascent proteomes in prion neurodegeneration

**DOI:** 10.1093/brain/awad313

**Published:** 2023-09-13

**Authors:** Hector Albert-Gasco, Heather L Smith, Beatriz Alvarez-Castelao, Dean Swinden, Mark Halliday, Sudha Janaki-Raman, Adrian J Butcher, Giovanna R Mallucci

**Affiliations:** UK Dementia Research Institute and Department of Clinical Neurosciences, University of Cambridge, Cambridge CB2 0AH, UK; Cambridge Institute of Science, Altos Labs, Great Abington CB21 6GP, UK; UK Dementia Research Institute and Department of Clinical Neurosciences, University of Cambridge, Cambridge CB2 0AH, UK; Cambridge Institute of Science, Altos Labs, Great Abington CB21 6GP, UK; Department of Biochemistry and Molecular Biology, Veterinary School, Complutense University of Madrid, 28040 Madrid, Spain; The San Carlos Hospital Health Research Institute, IdISSC, 28040 Madrid, Spain; UK Dementia Research Institute and Department of Clinical Neurosciences, University of Cambridge, Cambridge CB2 0AH, UK; Cambridge Institute of Science, Altos Labs, Great Abington CB21 6GP, UK; Cambridge Institute of Science, Altos Labs, Great Abington CB21 6GP, UK; Altos Labs Inc, Redwood City, CA 94065, USA; UK Dementia Research Institute and Department of Clinical Neurosciences, University of Cambridge, Cambridge CB2 0AH, UK; Cambridge Institute of Science, Altos Labs, Great Abington CB21 6GP, UK; UK Dementia Research Institute and Department of Clinical Neurosciences, University of Cambridge, Cambridge CB2 0AH, UK; Cambridge Institute of Science, Altos Labs, Great Abington CB21 6GP, UK

**Keywords:** neurodegeneration, UPR/ISR, nascent proteome, translational repression, trazodone, mitochondria, synapses

## Abstract

The unfolded protein response (UPR) is rapidly gaining momentum as a therapeutic target for protein misfolding neurodegenerative diseases, in which its overactivation results in sustained translational repression leading to synapse loss and neurodegeneration. In mouse models of these disorders, from Alzheimer’s to prion disease, modulation of the pathway—including by the licensed drug, trazodone—restores global protein synthesis rates with profound neuroprotective effects. However, the precise nature of the translational impairment, in particular the specific proteins affected in disease, and their response to therapeutic UPR modulation are poorly understood. We used non-canonical amino acid tagging (NCAT) to measure *de novo* protein synthesis in the brains of prion-diseased mice with and without trazodone treatment, in both whole hippocampus and cell-specifically.

During disease the predominant nascent proteome changes occur in synaptic, cytoskeletal and mitochondrial proteins in both hippocampal neurons and astrocytes. Remarkably, trazodone treatment for just 2 weeks largely restored the whole disease nascent proteome in the hippocampus to that of healthy, uninfected mice, predominantly with recovery of proteins involved in synaptic and mitochondrial function. In parallel, trazodone treatment restored the disease-associated decline in synapses and mitochondria and their function to wild-type levels.

In conclusion, this study increases our understanding of how translational repression contributes to neurodegeneration through synaptic and mitochondrial toxicity via depletion of key proteins essential for their function. Further, it provides new insights into the neuroprotective mechanisms of trazodone through reversal of this toxicity, relevant for the treatment of neurodegenerative diseases via translational modulation.

## Teaser

The antidepressant trazodone holds promise as a treatment for dementia. Albert-Gasco *et al.* explore the beneficial effects of trazodone in prion-diseased mice and find that the drug protects the brain via two main actions: maintaining the brain’s connections and restoring the function of energy-producing mitochondria.

## Introduction

Neurodegenerative diseases—including Alzheimer’s, Parkinson’s and related dementias—are characterized not only by the accumulation in the brain of disease-specific misfolded proteins, but also by overactivation of the unfolded protein response (UPR), specifically its PERK (PKR-like endoplasmic reticulum kinase) branch.^1-5^ The UPR is a ubiquitous cellular mechanism, its three branches resulting in coordinated signalling cascades aimed at correcting the misfolded protein stress.^6,7^ As part of this response, activation of PERK leads to phosphorylation of its target, eukaryotic initiation factor 2 (eIF2) on its alpha subunit, which blocks translation at the level of initiation reducing global protein synthesis rates in cells. Only a few specific mRNAs escape this translational repression when eIF2α-P levels are high.^6,7^

In human brains, high levels of PERK-P and eIF2α-P deposition parallel the deposition of disease-associated misfolded proteins.^1-5^ While the effects of UPR activation in human disease are unknown, the preclinical data support both a role in pathogenesis and a target for therapy.^8-10^ Thus, in animal models of Alzheimer’s disease,^11-13^ Parkinson’s disease,^14-16^ amyotrophic lateral sclerosis (ALS),^17,18^ tauopathies^19,20^ and prion disease,^21-25^ chronic PERK pathway activation results in the sustained reduction of global protein synthesis rates associated with subsequent synapse loss and neurodegeneration.^14-17,19,21-26^ Both genetic^21,24^ and pharmacological^14-17,19,22,23,25,26^ modulation of PERK/eIF2α-P signalling in these mice show cognitive improvement and neuroprotection and recovery of protein synthesis rates in many models.^12,14-17,20^ In prion disease, this rescues cognitive and synaptic function, prevents synapse loss and neurodegeneration and increases survival.^19,21-25^ Furthermore, in healthy mice, protein synthesis is essential for learning and memory and reducing eIF2α-P signalling at the synapse boosts cognition in wild-type^27-30^ and aged mice.^31^ Given the preclinical data and the neuropathological findings in human disease, the pathway has become a highly attractive target for the development of new therapies for use across the spectrum of neurodegenerative disease and is a focus for drug discovery.^8^ Several compounds, including the small molecule ISRIB^32^ and related compounds restore depressed global translation rates due to high eIF2α-P levels without systemic toxicity in mice and are in development for possible clinical use.^33^ Amongst compounds acting in this way is the licensed antidepressant trazodone, which is highly neuroprotective in mouse models of prion disease,^22^ frontotemporal dementia^22,34^ and ALS.^18^ Trazodone’s action in UPR/integrated stress response (ISR) modulation was discovered through unbiased screens,^22^ although its precise mechanism of action in reversal of translational repression is not known.

However, despite compelling evidence for the neurotoxic effects of chronic translational repression (which include repression of elongation^35-39^ as well as initiation, amongst other factors) and for the neuroprotective effects of treatments restoring global protein synthesis rates in the brain, the precise nature of the translational decline in disease—and its rescue—are not well understood. Earlier bulk proteomic studies in prion-diseased mice showed changes relating to decreased calcium signalling,^40^ neuroinflammation and complement activation.^41^ Recently, the *de novo* proteomes in mouse models of tauopathy^42^ and the APP/PS1 Alzheimer’s model^43,44^ (both of which have impaired protein synthesis rates^11,19,35,36^) were analysed, which describe changes in ribosomal^42,43^ and mitochondrial and synaptic proteins during the course of disease in bulk tissue or hippocampal slices.^42-44^ The response of the nascent proteome to modulation of pathways regulating translation in these models was not examined. To better understand the specific dysregulated UPR-related changes in the protein landscape induced during prion neurodegeneration and in response to treatment, we also used non-canonical amino acid tagging (NCAT) to measure *de novo* protein synthesis^45,46^ in the brains of prion-diseased mice.^21^ We analysed changes in the nascent proteome *in vivo*, with and without trazodone treatment during prion-induced neurodegeneration, across the whole hippocampus and also cell-specifically, in neurons and astrocytes, respectively. We examined these changes both qualitatively *in situ*, using fluorescence tagging (fluorescent non-canonical amino acid tagging, FUNCAT) and quantitatively, using proteomics after bio-orthogonal tagging (bio-orthogonal non-canonical amino acid tagging, BONCAT). We found that the decline in protein synthesis during disease reflects, in particular, the significant reduction in many proteins critical for synaptic and mitochondrial function, with an increase in apoptosis signalling in neurons. Critically, these changes are reversed by trazodone treatment, which remarkably largely restores the entire diseased-brain nascent proteomes both in the bulk hippocampal tissue and cell-specifically in hippocampal neurons and astrocytes. In parallel, trazodone treatment had both structural and functional protective effects on synapses and mitochondria, consistent with the changes specific to protein synthesis, restoring reduced numbers of both mitochondria and synapses, and restoring impaired mitochondrial function essential for synaptic and neuronal health. These data bring further granularity and detailed understanding of the proteins affected by translational repression during neurodegeneration across these disorders. Furthermore, for the first time, they show the recovery of the affected proteins and their associated organellar functions in response to therapeutic modulation, supporting the use of drugs such as trazodone to modulate the neurotoxic effects of translational repression.

## Materials and methods

### Prion infection, pharmacological treatment and metabolic labelling

**Figure 1:**
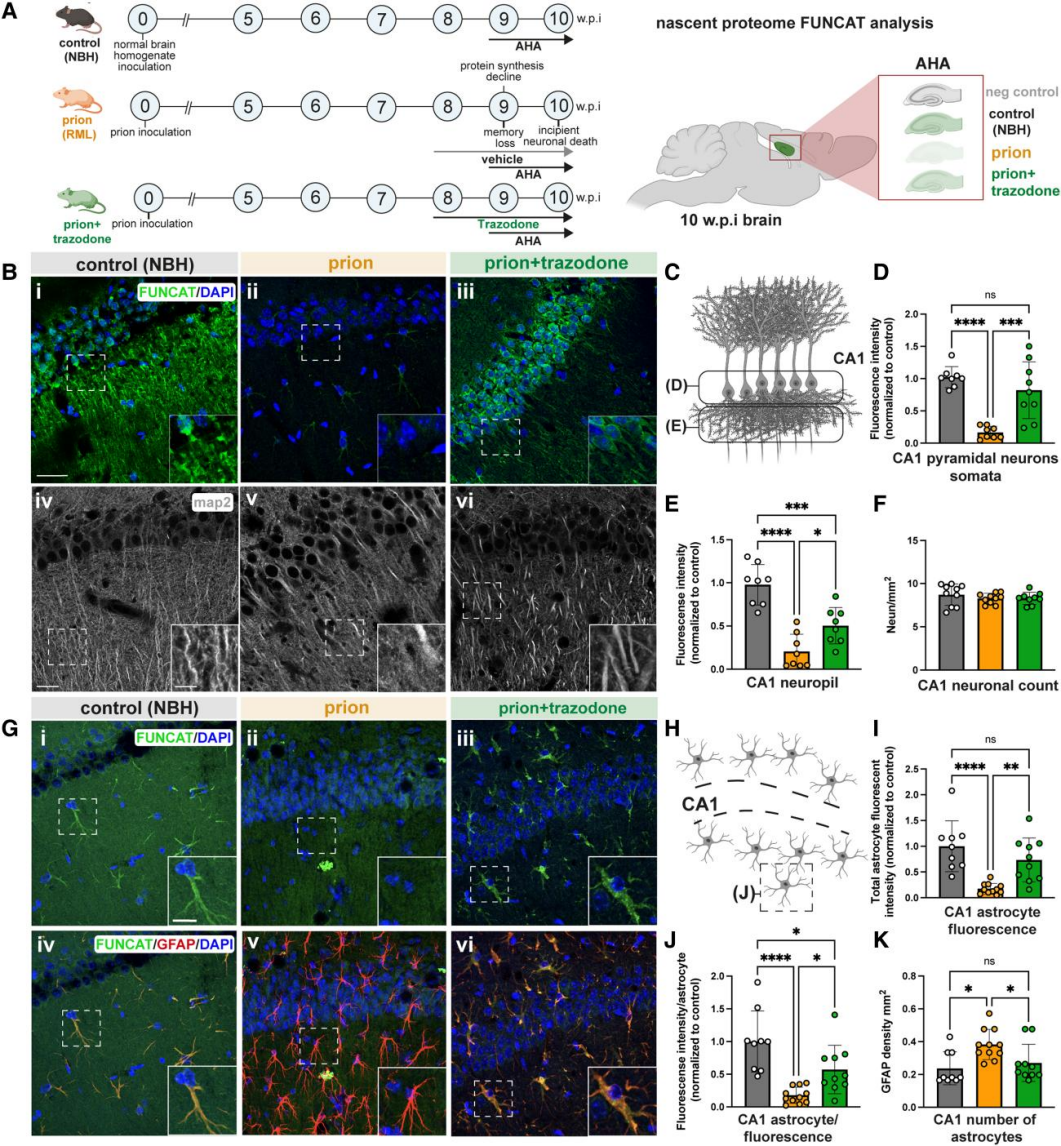
**Trazodone treatment restores repressed protein synthesis rates in prion-diseased hippocampal CA1 neurons and astrocytes**. (**A**) Schematic showing prion disease progression and timing of L-azidohomoalanine (AHA) labelling and trazodone treatment in tg37^+/−^ mice (*left*). Schematic of sagittal section of the mouse brain (*right*) showing hippocampal region analysed by fluorescent non-canonical amino acid tagging (FUNCAT). (**B**) Representative maximal projection images of the (**i**–**iii**) nascent neuronal proteome labelled by FUNCAT/DAPI from summated planes within stack (40 × 0.34 μm); and (**iv**–**vi**) MAP2-stained dendrites in CA1 for all conditions, showing marked reduction during prion disease and restoration by trazodone. (**C**) Schematic of the two regions of CA1 pyramidal neurons analysed. Fluorescence intensity quantification of newly synthesized proteins in (**D**) CA1 pyramidal neuron somata and (**E**) neuropil for all conditions calculated from summated stacks. (**F**) Neuronal counts in CA1 determined by NeuN staining and quantification. (**G**) Representative images of the (**i**–**iii**) nascent proteome in astrocytes labelled by FUNCAT/DAPI from single deep sections within 40 μm slice and (**iv**–**vi**) merged FUNCAT/GFAP/DAPI signal in control, prion and prion+trazodone, showing marked reduction during prion disease and restoration by trazodone. (**H**) Schematic showing location of CA1 astrocytes analysed. (**I**) Total fluorescence intensity for total CA1 astrocytes and (**J**) fluorescence intensity/astrocyte calculated from summated stacks for all conditions. (**K**) Numbers of astrocytes in CA1 region per condition. Scale bars in **B**(**i**) = 25 μm; **B**(**iv**) = 20 μm; **B**(**iv**, *inset*) = 5 μm and **G**(**i**, *inset*) = 10 μm. *P < 0.05, **P < 0.001, ***P < 0.0001, ****P < 0.00001. NBH = normal brain homogenate; ns = non-significant; w.p.i. = weeks post-inoculation.

Prion inoculation of tg37^+/−^ mice, NCAT::CamK2a and NCAT::GFAP transgenic mice (Jackson Laboratory, line #028071, #024098, 005359) was performed by inoculating 1% brain homogenate of Chandler/Rocky Mountain Laboratory (RML) prions, as described.^47^ Control animals for all strains of mice received 1% normal brain homogenate (NBH) and were culled at the same time points as prion and treated mice. Tg37^+/−^ prion disease mice were dosed intraperitoneally once daily with 40 mg/kg of trazodone hydrochloride (Merck T6154) or vehicle (saline)^22^ from 8 to 10 weeks post-inoculation (wpi), and from 16 to 18 wpi in NCAT::CamK2a and NCAT::GFAP mice (Fig. 1A and Supplementary Fig. 1B), as these mice have a slower prion disease progression. Mice were randomly assigned a treatment by cage number; no mice were excluded from the analysis.

We used non-canonical metabolic labelling to label the nascent proteome of controls (NBH), prion-diseased (prion) and prion-diseased trazodone treated (prion+trazodone) mice. Methionine surrogates L-azidohomoalanine (AHA) and L-azidonorleucine (ANL), which allow us to label nascent proteins in either non-cell specific or cell-specific manner, respectively, were used. Tg37^+/−^ whole hippocampus non-cell specific nascent proteome labelling was accomplished with 4 mM AHA (Fluorochem, # 942518–29-8) mixed with 5% maltose (Sigma, M9171) in drinking water and in a mash diet from 9 to 10 wpi. At 10 wpi prior to incipient neuronal death,^21^ all animals were sacrificed. Cell-specific nascent proteomes were generated via expression of a transgene in NCAT mice containing a point mutated MetRS* at L274G which allows the incorporation of ANL onto the mutated met-tRNA (Supplementary Fig. 1A). ANL (Iris Biotech, #HAA1625) was administered as a 1% solution in drinking water and mash diet.^45^ Mice were then culled at 18 wpi prior to incipient neuronal death.^21^ In parallel all groups had negative ANL/AHA controls to assess methionine surrogate incorporation.

### FUNCAT and immunofluorescence analysis

Neuronal death in tg37^+/−^ mice begins at 10 wpi and occurs mainly in the hippocampus, specifically in the CA1 region.^21^ Given this, we focused the FUNCAT analysis of the nascent proteome in the CA1 region of the hippocampus.

After metabolic labelling mice were perfused with PBS supplemented with 20 mM methionine. Fixed brains were then rinsed in PBS and stored at 4°C until slicing. Brains were mounted on 4% agar, sliced at 40 μm using a vibratome (Leica), incubated overnight with 0.5% Triton X-100 in PBS, washed extensively with PBS pH 7.8 and clicked overnight with Alexa Fluor™ 647 Alkyne (Thermo Fisher, A10278), triazole ligand (Sigma, # 678937), TCEP (Sigma, #C4706) and copper sulphate (Sigma, #C1297). This was followed by a brief rinse with PBS and mounting with DAPI mounting media. FUNCAT slices were always ‘clicked’ in parallel with negative AHA/ANL control slices. AHA/ANL negative and AHA positive unclicked slices did not show any FUNCAT signal (Supplementary Fig. 1E). After clicking, slices were extensively rinsed and incubated overnight with anti-GFAP (Abcam, #ab4674), anti-MAP2 (Invitrogen, #13-1500) or anti-NeuN (Abcam, ab177487) antibodies. On the next day slices were incubated with appropriate secondary fluorescent antibodies (Thermo Fisher). After rinsing, slices were mounted on plus-slides (Thermo Fisher), with anti-fade DAPI mounting media (Abcam, ab104139) and imaged using a Leica Stellaris SP8 confocal microscope. *Z*-stack images were taken using a 63× objective with 40 sections at 0.34 μm distance/section. *Z*-stacks were then combined summing fluorescence intensity for each of the sections to a single image.

To measure fluorescence intensity images were analysed with FIJI^48^ by subtracting from the overall intensity the product of the background intensity and total area analysed. CA1 fluorescent intensity at somas was segmented using DAPI and NeuN stain as a reference for hippocampal pyramidal neurons. CA1 fluorescence intensity signal from astrocytes was segmented using GFAP astrocyte specific marker as a reference (Supplementary Fig. 1D).

### BONCAT and nascent proteome isolation

For BONCAT analysis, ANL- and AHA-labelled hippocampi were dissected from brains. Dissected tissue was then homogenized and lysed in PBS pH 7.4 supplemented with 1% (w/v) Triton-100 and 0.8% (w/v) SDS, protease inhibitors (PI, 1:4000 dilution of protease inhibitor cocktail 3 without EDTA, Calbiochem, #539134) and benzonase (1:1000, Sigma) at 75°C for 15 min. Lysates were then cleared by centrifugation at room temperature for 20 min and stored at −80°C until further analysis. BONCAT was performed as previously described.^45^ In brief lysates were alkylated (iodoacetamide, Sigma, # I1149) for 4 h at room temperature. Following alkylation samples were clean-filtered (PD spintrap G-25 columns, GE Healthcare) and 32 μg of protein was clicked to 31 μM biotin-alkyne (Jena biosciences, #CLK-TA105) mixed in PBS pH 7.8-PI (Calbiochem, 1:4000), 300 μM Triazol ligand (Sigma, #678937) and 84 μg/ml CuBr (prepared by a 10 mg/ml dilution in DMSO, Sigma, 254185) at room temperature in the dark overnight.

All biotinylated proteins were then separated using an SDS-PAGE electrophoresis and immunoblotting with anti-biotin (Cell Signaling, #5597) and anti-GAPDH (Santa Cruz Biotechnology, #sc-32233) as loading control. This was followed by the incubation with secondary antibodies Starbright Blue 520 Goat Anti-Mouse (Bio-Rad, #12005866) and StarBright Blue 700 Goat Anti-Rabbit (Bio-Rad, # 12004161). BONCAT signal for all experimental groups was compared and normalized to their internal AHA/ANL clicked negative controls.

To determine the nature of each of the nascent proteomes, representative mice of each of the AHA/ANL-labelled mice cohorts with signal above that of negative controls were chosen for tandem mass spectrometry (LC-MS/MS) analysis. Alkylated and cleaned samples of AHA/ANL incorporated samples were clicked to a disulphide-biotin alkyne (DST) (Click Chemistry Tools, #1498) as described before. Optimal dose of DST alkyne was determined in each case (15–25 μM) before upscaling the reactions. Optimal DST alkyne dosages were then used to determine optimal Neutravidin bead (Pierce, #29200) dose for each case (9–15 μl dry beads). Upscaling using both optimal dosages of 1200 μg of lysate protein was affinity purified for each of the nascent proteomes incubating beads and clicked in PBS pH 7.4 with 0.1% SDS, 1% Triton X-100 and PI 1:1000 overnight at 4°C.

Following incubation, beads were extensively rinsed with Neutravidin wash buffer (PBS pH 7.4 1%, Triton X-100, 0.2% SDS + PI 1:1000), PBS + PI (1:1000) and 50 mM ammonium bicarbonate + PI (1:1000). Following bead rinses, each specific nascent proteome was eluted in 80% volume of dry beads in 5% β-mercaptoethanol, 0.03% SDS in 50 mM ammonium bicarbonate + PI (1:1000) mixed in water.

Following elution, quality of the elution was assessed by loading 50 μl of the resulting elution on a precast 4–15% gradient gel (Merk, MP41G15) and incubated with SYPRO RUBY (Merck, S4942) protein gel stain at room temperature. On the following day, gels were developed according to manufacturer’s instructions (Supplementary Fig. 2A). Once the ANL/AHA elutions were seen to be enriched compared to negative controls the rest of the elution was loaded on to gradient gels and sent for LC-MS/MS analysis.

Gel pieces were reduced (DTT) and alkylated (iodoacetamide) and subjected to enzymatic digestion with sequencing grade trypsin (Promega) overnight at 37°C. After digestion, the supernatant was pipetted into a sample vial and loaded onto an autosampler for automated LC-MS/MS analysis.

All LC-MS/MS experiments were performed using a Dionex Ultimate 3000 RSLC nanoUPLC (Thermo Fisher Scientific) system and a Q Exactive Orbitrap mass spectrometer (Thermo Fisher Scientific). Separation of peptides was performed by reverse-phase chromatography at a flow rate of 300 nl/min and a Thermo Scientific reverse-phase nano Easy-spray column (Thermo Scientific PepMap C18, 2 mm particle size, 100A pore size, 75 mm i.d. × 50 cm length). Peptides were loaded onto a precolumn (Thermo Scientific PepMap 100 C18, 5 mm particle size, 100A pore size, 300 mm i.d. × 5 mm length) from the Ultimate 3000 autosampler with 0.1% formic acid for 3 min at a flow rate of 10 ml/min. After this period, the column valve was switched to allow elution of peptides from the precolumn onto the analytical column. Solvent A was water + 0.1% formic acid and solvent B was 80% acetonitrile, 20% water + 0.1% formic acid. The linear gradient employed was 2–40% solvent B for 30 min. Further wash and equilibration steps gave a total run time of 60 min.

The LC eluent was sprayed into the mass spectrometer by means of an Easy-Spray source (Thermo Fisher Scientific). All *m*/*z* values of eluting ions were measured in an Orbitrap mass analyzer, set at a resolution of 35 000 and was scanned between *m*/*z* 380–1500. Data-dependent scans (Top 20) were employed to automatically isolate and generate fragment ions by higher energy collisional dissociation (HCD, NCE:25%) in the HCD collision cell and measurement of the resulting fragment ions was performed in the Orbitrap analyser, set at a resolution of 17 500. Singly charged ions and ions with unassigned charge states were excluded from being selected for MS/MS and a dynamic exclusion window of 20 s was employed.

### Analysis of LC-MS/MS data

LC-MS/MS raw data were processed using MaxQuant (Max Planck Institute), matching spectra to the *Mus musculus* database from UniProt (Tax ID 10090). Peptide identifications were calculated with false discovery rate (FDR) < 0.01 for multiple comparisons (see further parameters in Supplementary Table 1) and proteins with more than two peptides were taken further for the analysis. Relative normalized abundance intensity of proteins was given by a label-free quantification approach (LFQ).

Resultant identified proteins peptide intensities were then filtered only for those proteins with higher LFQ values than negative control (Supplementary Fig. 2B). Cases in which LFQ values were lower than negative controls were changed to 0. This was followed by a differential expression analysis done using R package DEP^49^ (Bioconductor, 1.19.0). All nascent proteomes were first normalized and imputed (MinProb, q = 0.01). Proteins were considered differentially expressed if α < 0.05. Hierarchical mappings with dendrograms clustering both LFQ Log2 normalized intensities and mice as well as principal component analysis (PCA) plots were generated using the DEP package visualization tool. Volcano plots comparing two sets of nascent proteomes were generated using the output DEP results and with Graph-pad prism 9 (all contrasts available in Supplementary Table 2).

Following differential protein expression analysis, significantly differentially expressed proteins from all nascent proteomes were used for Ingenuity Pathway Analysis (IPA) (Qiagen) to highlight activated/inhibited pathways when comparing control (NBH) versus prion, control (NBH) versus prion + trazodone and prion + trazodone versus prion. Representative heat maps of canonical pathways which were significant and showed predicted activation/inhibition of the pathway were represented using the *z*-score.

### Immunoblotting

After identification of the different nascent proteomes, validation of protein changes were performed by immunoblotting. Synaptogenesis pathways was validated by loading of 10 μg of protein in 4–20% gradient gels from 18 wpi NCAT::CamK2a mice from each treatment group, and blotted for synaptophysin (Cell Signaling, 36406) and vesicle-associated membrane protein 2 (Cell Signaling, 13508) levels. GAPDH (Santacruz, sc-32233) was used as a loading control.

Oxidative phosphorylation was validated by loading 10 μg of hippocampal protein lysate from 18 wpi NCAT::CamK2a mice from the three experimental groups and immunoblotting for an oxidative phosphorylation (OXPHOS) mouse cocktail (Abcam, ab110413). Β-actin (Abcam, ab8227) was used as loading control.

### Electron microscopy

Synapse and mitochondrial numbers were quantified using scanning electron microscopy (SEM) of CA1 hippocampal pyramidal neurons. Prion tg37^+/−^ mice treated with trazodone from 8 to 10 wpi were compared with prion only and control (NBH) animals (*n* = 3), were transcardially perfused with 2% paraformaldehyde (PFA), 2% glutaraldehyde in 0.9% saline. Perfusions were followed by 24 h post-fixation at 4^ο^C and incubated in sodium cacodylate for 4 days. Afterwards, brains were embedded in 4% agar and sliced to 300 μM using a vibratome (Leica), osmosized and re-sliced using an ultramicrotome for SEM imaging. The imaging of the CA1 was divided into two regions of interest, first we imaged CA1 at the pyramidal cell layer to include normally degenerating neuronal somas. Second, we imaged synapses at the CA1 stratum radiatum.

Areas of 20 mm^2^ were assessed for mitochondrial numbers and 10 mm^2^ for synaptic numbers. Mitochondrial counts have been expressed as the number of mitochondria per pyramidal neurons counted, while synapses counts were expressed per 55 μm^2^ to compare with previous studies.^26,50^ Mitochondria were considered present within synapses when found in either a dendrite or an axon at a synapse, expressed per 100 μm^2^.

### Mitochondrial isolation and mitochondrial stress test

To assess mitochondrial respiration, we used mitochondrial isolation combined with a mitochondrial stress test on a Seahorse XF96 Analyzer (Agilent). Isolation of the mitochondria and the mitochondrial stress test followed previously published methods with slight modifications adapted to our specific tissue.^51^

We isolated mitochondria from control (NBH), prion and prion + trazodone hippocampi from mice treated from 8 to 10 wpi. Each animal was a biological replicate. To isolate hippocampal mitochondria we first dissociated both hippocampi from each animal using mitochondrial isolation buffer [MIB1: 210 mM of D-mannitol, 70 mM of sucrose, 5 mM of HEPES, 1 mM of EGTA and 0.5% (w/v) of fatty acid-free BSA pH 7.2] using 15 to 20 stocks with a manual Potter-Elvehjem PTFE pestle (Sigma) using a 8 ml glass tube until tissue fully homogenized. This was followed by a series of centrifugations to resolve the mitochondrial fraction from the parts of the cell (800*g*, 8000*g*). Once isolated we rinsed the mitochondrial fraction twice with MIB1 and then resuspended in mitochondrial isolation buffer [MAB1: 220 mM of D-mannitol, 70 mM of sucrose, 10 mM of KH_2_PO_4_, 5 mM of MgCl_2_, 2 mM of HEPES, 1 mM of EGTA and 0.2% (w/v) of fatty acid-free BSA, pH 7.2]. Once ready for testing we quantified the amount of mitochondria from each isolation using Bio-Rad Protein Assay Kit (Bio-Rad, #5000001), plated 3 μg/50 μl/well onto a 96-well plate with all animals having 6–8 technical replicates (101085-004, Agilent). Mitochondrial plates were then centrifuged at 2000*g*, 4°C, for 20 min. After centrifugation we incubated the mitochondrial-plate in a non-CO_2_ incubator at 37°C for 10 min before running the mitochondrial stress test.

The mitochondrial response to the differing stressors was assessed using an established protocol.^51^ In brief, we first measured basal respiration rate in the presence of all substrates (10 mM succinate, 5 mM malate and 10 mM glutamate), followed by injection of 4 mM ADP (Sigma, A2754), 5 μM oligomycin (Sigma, O4876), 5 μM FCCP (Sigma, C2920) and 5 μM rotenone + antimycin A (Sigma, R8875, A8674). As a quality control of the isolated mitochondria we stained with 200 μM MitoTracker^®^ Red CMXRos mixed in MAB1 (Cell Signaling, #9082) showing intact mitochondria for all conditions (Supplementary Fig. 4D). Basal respiration was calculated by subtracting basal oxygen consumption rate (OCR) minus the OCR after injection of rotenone and antimycin A. Respiration after ADP injection was calculated as the subtraction of ADP OCR minus the OCR after injection of rotenone and antimycin A. Proton leak respiration was calculated as the OCR after oligomycin injection minus the OCR after injection of rotenone + antimycin A. Maximal respiratory capacity was measured as the OCR value after FCCP injection minus the OCR after rotenone + antimycin A injection.

### Statistical analysis

Statistical analysis of FUNCAT and BONCAT analysis to assess AHA/ANL incorporation was done using a one-way ANOVA followed by Tukey’s multiple comparison test with an α of 0.05, analysed using Graph pad prism 9. Differential expression analysis on nascent proteomes was done using Stats and limma (Bioconductor 3.17), with R packages contained within the DEP package.^49^ Statistical analysis of significantly enriched pathways (*P* < 0.05) from significant proteins was obtained from IPA. Significant analysis of interacting clusters was obtained through metascape-String analysis.^52^ Statistical analysis of quantitative changes in synaptic proteins and OXPHOS components on immunoblots; synapse and mitochondrial counts by SEM; and differences in mitochondrial respiration were each performed using a one-way ANOVA followed by a Tukey multiple comparison test with an α of 0.05 analysed using Graph pad prism 9.

## Results

### FUNCAT: trazodone restores global protein synthesis rates in prion-diseased neurons and astrocytes

We used tg37^+/−^ mice^47^ inoculated with RML prions for whole hippocampal analyses (see schematic in Fig. 1A). These transgenic mice, used extensively in our previous studies,^21-25,47,50,53^ overexpress prion protein (PrP) ∼3-fold and have a relatively rapid disease course, dying at 12 wpi. They show onset of UPR activation and protein synthesis rates decline from 9 wpi, along with cognitive impairment and behavioural change and synaptic dysfunction, with onset of neuronal death in the hippocampal CA1–3 region beginning at 10 wpi, followed by overt neurodegeneration and clinical signs of prion disease and death at 11–12 wpi.^21^ We used NCAT to determine the changes in the nascent proteome in whole tissue due to UPR activation and its response to treatment, to better understand the specific processes leading to neuronal death in prion neurodegeneration. Thus, the methionine analogue AHA, when incorporated into proteins, allows either fluorescence or biotin labelling of AHA-containing proteins through click chemistry. Fluorescence-AHA-labelled proteins can be visualized using FUNCAT, while biotin-AHA-labelled proteins can be affinity purified and further analysed using BONCAT^45,46^ (see schematic in Supplementary Fig. 3A). We first visualized global protein synthesis rates, using FUNCAT, in hippocampal sections from prion-infected tg37^+/−^ mice treated with vehicle or trazodone from 8 wpi, and a control group inoculated with NBH. AHA was administered between 9 and 10 wpi in all groups to ensure widespread incorporation during the period of UPR activation and translational repression that leads to incipient neuronal loss (Fig. 1A). Animals were sacrificed at 10 wpi for analysis and their hippocampi examined. We found a dramatic decline in global protein synthesis rates in the CA1 region pyramidal neuron cell bodies of prion-diseased mice, with an average fluorescence intensity (FI) of 0.17, compared to a FI of 1.02 in uninfected controls (*P* < 0.0001) [Fig. 1B(i and ii) and D]. This decline was also seen in neurites: FI 0.2 in prion versus FI 0.98 in controls (*P* < 0.0001) [Fig. 1B(i and ii)]. Remarkably, treatment with trazodone restored repressed protein synthesis rates almost completely in neuronal cell bodies: FI 0.82 versus 0.17 untreated (*P =* 0.0003) and partially in the neuropil: FI 0.5 versus 0.17 in untreated (*P* = 0.0301) [Fig. 1B(i–iii), D and E]. The reduction—and restoration—of protein synthesis rates was not due to changes in neurite density in the different conditions, as confirmed by comparable levels of NeuN/MAP2 labelling in neurites in all experimental groups [Fig. 1B(iv–vi)], nor to changes in CA1 pyramidal neuron density (Fig. 1F).

Astrocytes are also affected by prion disease, showing marked proliferation and activation^24,53,54^ (Fig. 1G and I–K). Indeed, astrocytic UPR activation leads to a reactive state that contributes to neurodegeneration via reduced trophic support to neurons via an altered secretome resulting from reduced translation rates.^24^ Co-labelling with FUNCAT and the astrocytic marker, GFAP, in CA1 sections revealed marked repression of the nascent proteome in astrocytes in prion-disease, consistent with our previous findings,^24^ which was restored by treatment with trazodone [Fig. 1G(i–vi)]. Fluorescent intensity quantification (Fig. 1H) confirmed a lowered rate of nascent protein synthesis in prion disease versus control, both per astrocyte: FI 0.18 versus 1.00 (*P* < 0.0001) and for all astrocytes: FI 0.17 versus 1.00; (*P* < 0.0001), respectively (Fig. 1I and J), which was largely restored by trazodone treatment: FI 0.75/astrocyte versus FI 1.00 in controls (*P* = 0.0352) and FI 0.73 for all astrocytes versus FI 1.00 in controls; *P =* 0.005) (Fig. 1I and J). In untreated mice, the decline of the fluorescence intensity signal occurred despite a significant increase in density of astrocytes, consistent with reduced translation rates in activated diseased astrocytes (Fig. 1K), again in agreement with previous findings.^24^

In parallel, we performed FUNCAT on mice in which we labelled the nascent proteome in a cell-specific manner. We used NCAT-mice,^46^ which, when crossed with specific Cre-expressing lines, in this case CamK2a-Cre and GFAP-Cre, express mutated methionine tRNA synthetase, MetRS*, in forebrain neurons and astrocytes, respectively (Supplementary Fig. 1A). This protein will only incorporate the methionine analogue, azidonorleucine (ANL), into new proteins, similar to AHA. NCAT::CamK2a and NCAT::GFAP mice were inoculated with RML prions and treated either with trazodone or vehicle, as before. Controls were inoculated with NBH (Supplementary Fig. 1B). NCAT and the Cre lines do not overexpress PrP and thus have a slower incubation period than tg37^+/−^ mice, typical for wild-type C57/Bl6 mice. Accordingly, UPR activation and translational repression occurs at 17 wpi, with neuronal loss beginning around 18 wpi and terminal clinical signs at 22 wpi.^21^ Consistent with this, FUNCAT-labelling with ANL of the CamK2a-specific nascent proteome from 17 to 18 wpi confirmed marked repression of protein synthesis rates in CA1 pyramidal neurons, which was restored by trazodone treatment [Supplementary Fig. 1B(i–iii)], equivalent to the changes seen in tg37^+/−^ mice at 9 to 10 wpi [Fig. 1B(i–iii)]. Similarly, FUNCAT-labelling with ANL of the astrocyte-specific nascent proteome showed repression in global protein synthesis rates at 17 wpi in prion-diseased CA1 astrocytes and its restoration with trazodone treatment at 18 wpi [Supplementary Fig. 1C(iv–vi)]. [Background fluorescent signal due to ‘click’ effect was negligible for all experimental groups (Supplementary Fig. 1E(i–vi).] ANL-labelling of proteins in the nascent proteome detected by FUNCAT or BONCAT differs only in the nature of the clicked ligand (see Supplementary Fig. 3A for schematic), fluorescent 647-alkyne versus Biotin-DST-alkyne, respectively, binding to ANL, so cell specificity defined by FUNCAT (Supplementary Fig. 1B and C) applies also to the BONCAT samples analysed, see below.

### BONCAT: trazodone restores nascent proteomes in prion-diseased neurons, astrocytes and hippocampus

**Figure 2:**
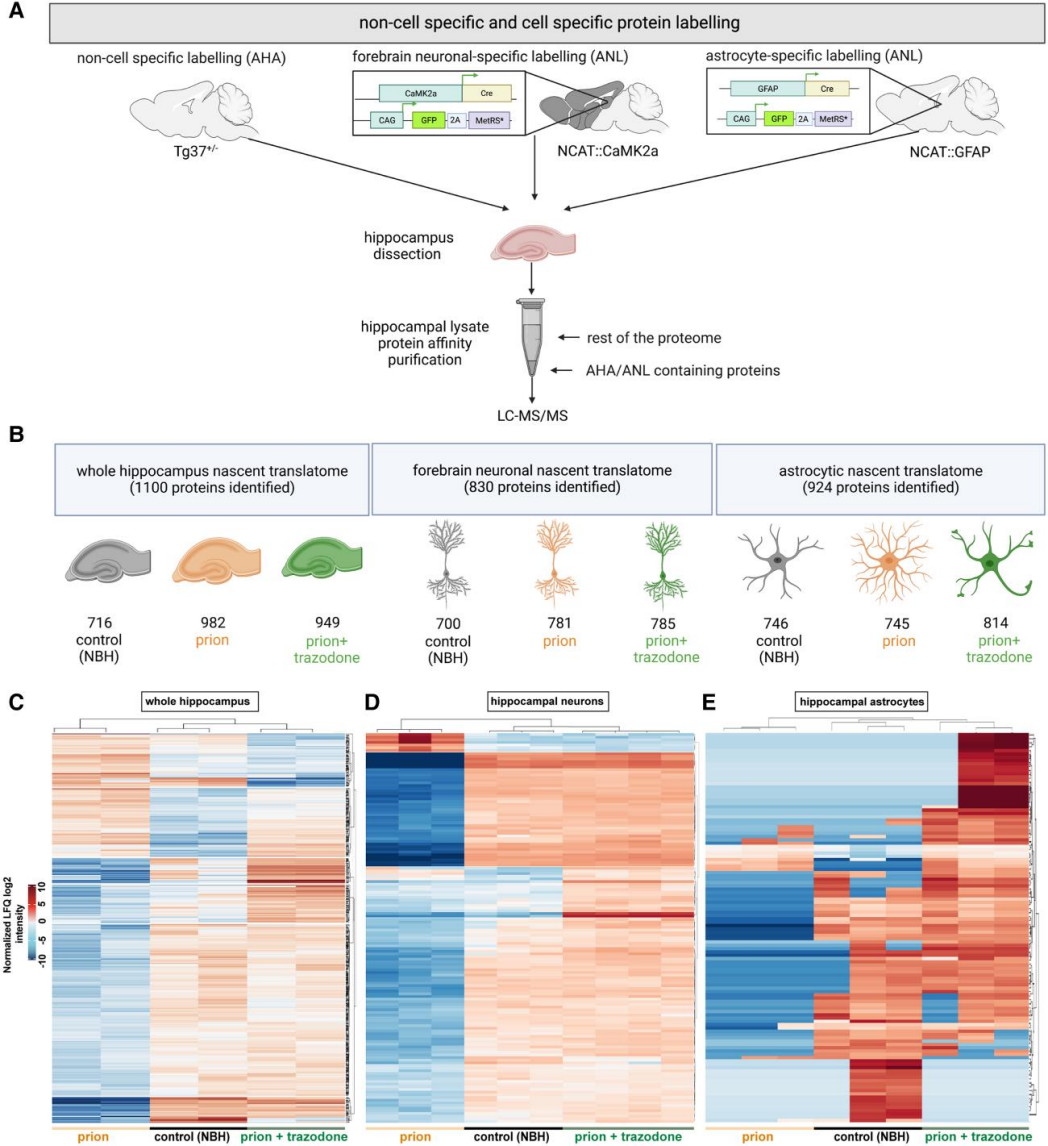
**Trazodone restores the nascent proteomes of neurons, astrocytes and whole hippocampus in prion-diseased brains.** (**A**) Schematic showing experimental steps for bioorthogonal non-canonical amino acid tagging (BONCAT) analysis: and L-azidohomoalanine (AHA)/L-azidonorleucine (ANL) labelling of the mouse brain, hippocampal dissection, followed by affinity purification of AHA/ANL-labelled proteins. (**B**) Total numbers of proteins detected in control [normal brain homogenate (NBH)], prion and prion + trazodone treatment groups by liquid chromatography-tandem mass spectrometry in the whole hippocampus, in neurons and in astrocytes, respectively. (**C**) Hierarchical heat map by log2 normalized label-free quantification (LFQ) intensity from whole hippocampus, (**D**) neurons and (**E**) astrocytes, showing clustering of proteins and mice per experimental group. Differentially expressed significant proteins, *P* < 0.05.

We next used BONCAT to isolate the individual nascent proteomes followed by LC-MS/MS from AHA-labelled whole hippocampus (tg37^+/−^ mice) and ANL-labelled hippocampal neurons (NCAT::CamK2a mice) and astrocytes (NCAT::GFAP mice) for each of the three experimental conditions (NBH control, prion-diseased, prion-disease + trazodone treatment) (see schematic in Fig. 2A). All nascent proteomes had an average signal above background and confirmed findings from FUNCAT analysis (Supplementary Fig. 2). We identified a total of 1100 proteins in the nascent proteomes of whole hippocampi from tg37^+/−^ mice, of which 716 were in control samples, 982 in prion-only and 949 in prion + trazodone samples. In the neuron-specific nascent proteomes, we identified 830 proteins of which 700 were in controls, 781 in prion-only and 785 in prion + trazodone treated mice. In the astrocyte-specific nascent proteomes there were 924 proteins of which 746 proteins were identified in controls, 745 proteins in prion-only and 814 in prion + trazodone samples (Fig. 2B; full list in Supplementary Table 2). BONCAT quality control on elutions was confirmed by Sypro Ruby protein staining and log2 intensity enrichment plots (Supplementary Fig. 2A and B). Further, in addition to FUNCAT verification of cell-specificity (Supplementary Fig. 1B–D) the neuronal and astrocytic nascent proteomes were positive for their corresponding cell-specific markers (Supplementary Fig. 2C), and the whole hippocampal nascent proteome included markers of oligodendrocytes, microglia and endothelial cells (Supplementary Table 2).

**Figure 3:**
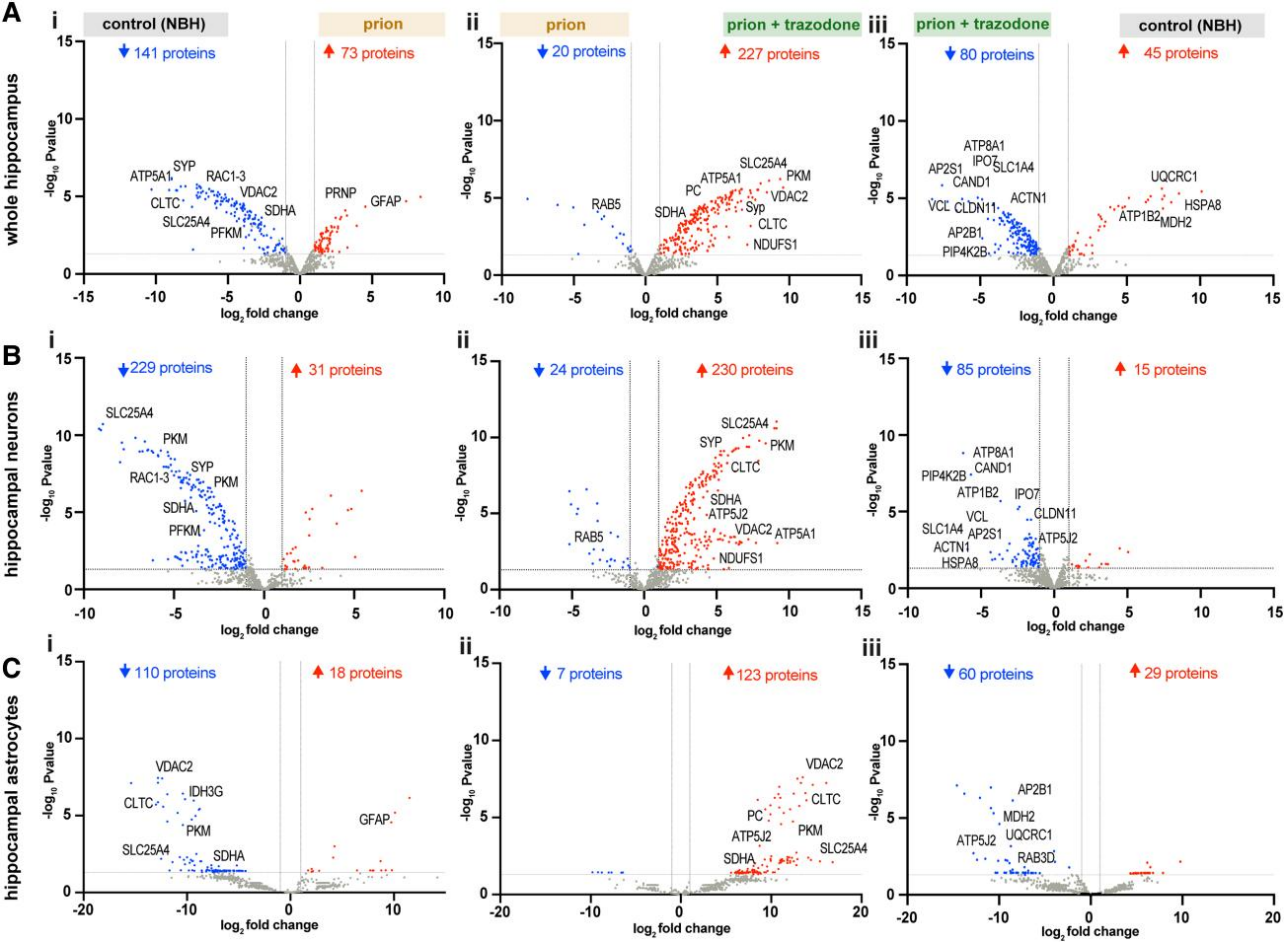
**Trazodone treatment restores specific subsets of proteins in the whole hippocampus and cell-specifically, in neurons and astrocytes**. (**A**) Volcano plots of whole hippocampus nascent proteomes comparing (**i**) prion with control [normal brain homogenate (NBH)] nascent proteomes; (**ii**) prion + trazodone with prion; and (**iii**) control (NBH) with prion + trazodone. (**B**) Volcano plots of hippocampal forebrain neuronal nascent proteomes comparing (**i**) prion with control (NBH) nascent proteomes; (**ii**) prion + trazodone with prion; and (**iii**) control (NBH) with prion + trazodone. (**C**) Volcano plots of hippocampal astrocytic nascent proteomes comparing prion with (**i**) control (NBH) nascent proteomes; (**ii**) prion + trazodone with prion; and (**iii**) control (NBH) with prion + trazodone. Differentially expressed significant proteins, *P* < 0.05.

**Figure 4:**
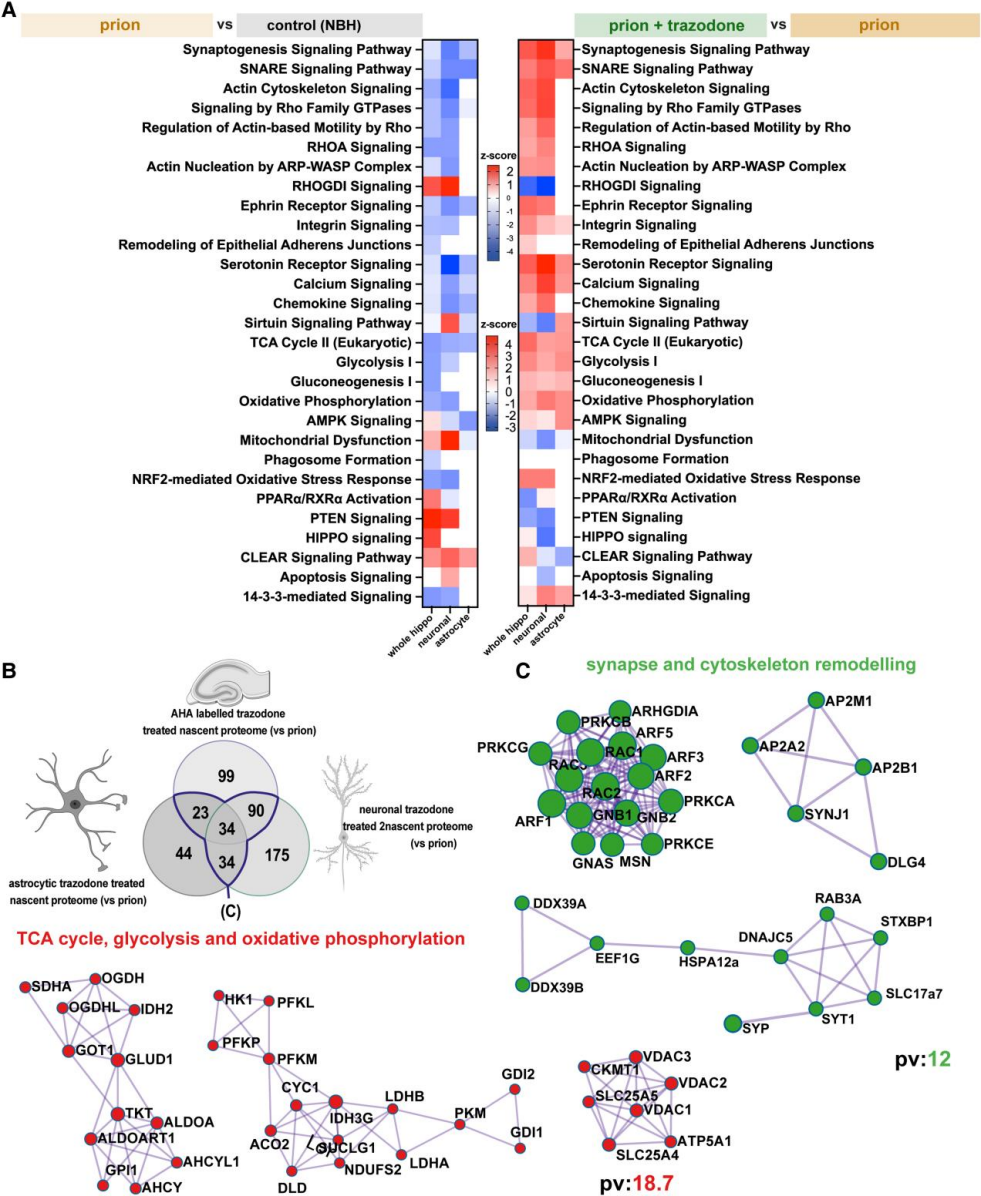
**Synapse remodelling and mitochondrial function are the predominantly dysregulated pathways in prion disease and are restored by trazodone treatment.** (**A**) Heat maps of significant pathways showing differences in *z*-score of prion (orange) versus control [normal brain homogenate (NBH)] (grey) nascent proteomes (*left*), and heat maps of significant pathways showing differences in *z*-score of prion + trazodone (green) versus prion (orange) nascent proteomes (*right*). (**B**) Venn diagram of overlapping prion + trazodone nascent proteomes from whole hippocampus, neurons and astrocytes, compared to prion alone (blue line highlights overlap). (**C**) Significant clusters of overlapping prion + trazodone nascent proteomes, determined by metascape-string analysis, of significantly upregulated proteins compared to prion alone. The proteins clustered to synapse and cytoskeleton remodelling, and to the TCA cycle, glycolysis and oxidative phosphorylation. *P* < 0.05 for significant proteins. pv = −log10*P*-value.

We used LFQ values to analyse relative differences in abundance of proteins between the different nascent proteomes. When represented in hierarchical heat maps, LFQ normalized values of significantly expressed proteins showed clustering of values from control, prion and prion + trazodone animals for each condition (Fig. 2C–E). Prion disease caused overall reduction (blue cells) in many proteins across each condition compared to high levels (red cells) in control animals. Heat maps from trazodone-treated animals were very similar to those of controls across each condition, with the majority of proteins restored to control levels, other than a small proportion (Figs 3 and 4 and Supplementary Table 2). The astrocytic nascent proteome showed more variability in disease versus control and trazodone-treated mice, reflecting the phenotypic variability and mixed profiles derived from varying activation states of astrocytes in disease,^55,56^ but the groups still cluster as the others (Fig. 2D). In line with these data, analysis of variance by PCA, control and prion + trazodone cluster separately to the nascent proteomes of untreated prion-diseased animals for both the whole hippocampus and hippocampal neurons, but are less clear-cut for astrocytes (Supplementary Fig. 3D). Again, we attribute this to variability in astrocytic states, which is well documented in health and particularly in disease, and not to batch effects, as all samples cluster within experimental groups.

Volcano plots comparing the nascent proteomes for prion-disease versus controls for whole hippocampi showed 141 proteins were significantly downregulated and 73 upregulated (Fig. 3A). For the CamK2a-specific dataset, 229 proteins were downregulated and 31 were upregulated (Fig. 3B), and in the astrocyte-specific dataset, 110 proteins were downregulated and 18 were upregulated (Fig. 3C). In contrast, when comparing the nascent proteomes of prion + trazodone treatment to prion-only, the situation is reversed, with upregulation of 227, 230 and 123 proteins in whole hippocampi, neurons and astrocytes, respectively, and downregulation of 20, 24 and 7, respectively (Fig. 3A–C). Altogether, the comparison of trazodone-treated compared to prion-diseased nascent proteomes highlights recovery of the overall landscape of the diseased brain across different cell types. Consistent with this, the nascent proteomes of controls versus trazodone-treatment are relatively similar across the three groups with 45, 15 and 29 proteins upregulated and 80, 85 and 60 downregulated (Fig. 3A–C). These data are particularly compelling given the consistency between different mouse models, the tg37^+/−^ mice (FVB background, rapid disease course) and the NCAT::CamK2a and NCAT::GFAP mice (C57/Bl6 background, slower disease course), and between whole hippocampal nascent proteome from tg37^+/−^ mice with their mixed cell population and the neuron- and astrocyte-specific nascent proteomes from the NCAT mice.

Proteins consistently downregulated in prion disease compared to controls include those related to synapse formation and maintenance, such as SYP or RAC1, and to ATP biosynthesis, such as PKM, SDHA, VDAC2 SLC25A4, in all samples [Fig. 3A(i), B(i) and C(i)]. Trazodone treatment upregulated (restored) many of these, including SYP, SYNGR1, DLG4 and PKM, SDHA, VDAC2 and SLC25A4, and other mitochondrial proteins ATP5A1/J2, NDUFS1 or PC [Fig. 3A(ii), B(ii) and C(ii) and Supplementary Table 2]. Interestingly, some proteins including ATP-hydrolysis factors (ATP1B2, ATP8A1), amino acid importers (SLC1A4), cell adhesion proteins (CLDN11, VCL), mediators of autophagy (PIP4K2B) or protein ubiquitination (CAND1) were higher in trazodone-treated samples than controls, suggesting an overshoot or upregulation of these by trazodone treatment in disease [Fig. 3A(iii), B(iii) and C(iii)]. A few proteins upregulated in control compared to trazodone-treated whole hippocampi (HSPA8, ATP1B2, MDH2, UQCRC1) were downregulated in the cell-specific nascent proteomes (HSPA8, ATP1B2) in neurons and MDH2, UQCRC1 in astrocytes.

Taken together, these data highlight the profound impact of prion disease on the nascent proteome during early neurodegeneration seen at the level of both bulk hippocampal tissue as well as in hippocampal neurons and astrocytes, with profound impairment in synaptic and mitochondrial proteins, further defined below. Trazodone treatment restored many of these proteins, consistent with its known neuroprotective effects.

### Trazodone restores dysregulated pathways controlling synapse and mitochondrial function

We further analysed significantly differentially expressed proteins represented in the volcano plots (Fig. 3). Using their log2-fold change values, we applied IPA to reveal up- or downregulated canonical pathways for each of the nascent proteomes (Fig. 4A). Across all the prion versus control nascent proteomes, many important pathways related to key aspects of hippocampal function were downregulated, including synaptogenesis signalling and other synapse-related pathways—SNARE signalling, actin cytoskeleton signalling and others. Another major group of downregulated pathways related to mitochondrial function, including TCA cycle, glycolysis and OXPHOS, although the latter two are not downregulated in the astrocyte-specific nascent proteome. Upregulated pathways in prion-diseased animals versus controls include RHOGDI signalling—related to inhibition of actin Rho small GTPases—mitochondrial dysfunction and CLEAR signalling and in neurons specifically, apoptosis (Fig. 4A). These changes are reversed to varying degrees by trazodone treatment, both within the whole hippocampal nascent proteomes of prion-diseased tg37^+/−^ mice and within the neuron- and astrocyte-specific nascent proteomes of prion-diseased NCAT mice. In particular, these data from prion + trazodone versus prion highlight the restoration of synaptogenesis, SNARE and actin cytoskeleton signalling pathways as well as Rho GTPases, RHOA, ephrin, integrin and serotonin signalling, the latter in whole brain and neurons, consistent with changes in these nascent proteomes during disease. Trazodone treatment results in recovery also of mitochondrial pathways: glycolysis, TCA cycle and oxidative phosphorylation signalling in all nascent proteomes, with reduction of mitochondrial dysfunction and neuronal apoptosis (see full list in Supplementary Table 3).

We next looked at the specific proteins affected in all the trazodone-treated nascent proteomes compared to vehicle-treated nascent proteomes (Fig. 4B and full details in Supplementary Table 4) and analysed these by metascape-STRING^52^ (Fig. 4C). Consistent with the IPA analysis, this confirmed two main clusters of highly interacting proteins, one related to synapse and cytoskeleton remodelling and the other to mitochondrial biology: TCA cycle, glycolysis and OXPHOS (Fig. 4C).

### Quantitative and functional rescue of synapses and mitochondria by trazodone

**Figure 5:**
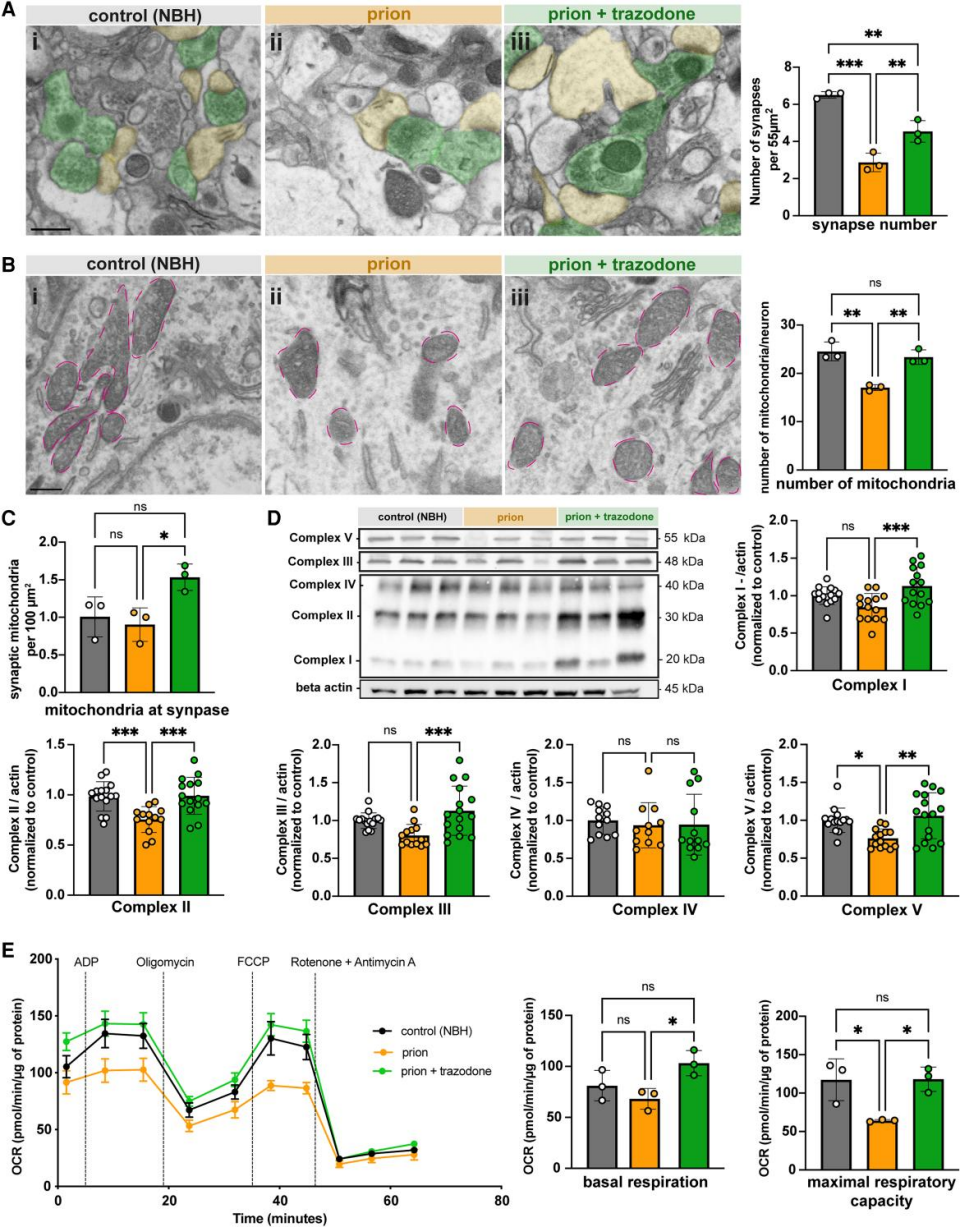
**Trazodone treatment restores synapse and mitochondrial numbers and function in prion disease.** (**A**) Representative scanning electron microscopy (SEM) images from (**i**) control [normal brain homogenate (NBH)]; (**ii**) prion; and (**iii**) prion + trazodone mice from CA1 stratum radiatum. Dendrites are pseudo-coloured in yellow and axons in green. Synapse number quantification of the hippocampal CA1 neuropil shows a decrease in prion disease (orange bars) and partial but significant restoration with trazodone treatment (green bars). (**B**) Representative SEM images from (**i**) control (NBH); (**ii**) prion; and (**iii**) prion + trazodone from CA1 pyramidal neuron somata, with mitochondria perimeter coloured in pink. The numbers of mitochondria/neuron are reduced in prion disease (orange bars) and restored to wild-type levels with trazodone treatment (green bars). (**C**) Mitochondria counted within synapses increase with trazodone treatment. (**D**) Immunoblots and quantification (bar graph, *right*) of oxidative phosphorylation proteins (I, II, III, IV and V) showing restoration with trazodone treatment. (**E**) Mitochondrial stress test on isolated mitochondria from control (NBH), prion and prion + trazodone treatment groups at 10 weeks post-inoculation (wpi) as before (Fig. 1A), showing oxygen consumption rates (OCRs) from 0 to 65 min, with time points of stressor addition (ADP, oligomycin, FCCP and rotenone + antimycin A, respectively) labelled. Basal respiration was defined as the (initial) OCR value minus (rotenone + antimycin A) OCR value; maximal respiratory capacity was defined as the OCR value after FCCP injection minus (rotenone + antimycin A) OCR. *P < 0.05, **P < 0.001, ***P < 0.0001. Scale bars in **A**(**i**) and **B**(**i**) = 1 μm.

For a morphological assessment of the proteomics results implicating synaptogenesis and mitochondrial pathway impairments, we used electron microscopy to assess synapse and mitochondrial numbers in the CA1 region in all groups of tg37^+/−^ (as per schematic in Fig. 1A). Synaptic density was dramatically lowered in prion-disease (2.8 synapses/55 μm^2^) compared to controls (6.5 synapses/55 μm^2^; *P* < 0.0002), consistent with previous findings.^21,50,57^ In contrast, 2 weeks of trazodone treatment significantly rescued synapse density (to 4.54 synapses/55 μm^2^; *P =* 0.0096), although not to wild-type levels (Fig. 5A). Synaptic proteins, detected by immunoblotting, also increased (Supplementary Fig. 4A). In our previous work, we showed that trazodone restores memory in prion-diseased mice,^22^ a functional recovery consistent with its rescue of synapse number and protein levels demonstrated here.

Mitochondrial numbers in the neuronal cell bodies were reduced in disease (17/pyramidal neuron) compared to controls (25/pyramidal neuron; *P =* 0.0018) and restored to wild-type levels by 2 weeks of trazodone treatment (23.3/pyramidal neuron; *P =* 0.0045) (Fig. 5B). Trazodone also restored mitochondria numbers within dendrites and axons of the CA1 neurons (Fig. 5C). In parallel, trazodone treatment produced significant recovery in oxidative phosphorylation protein complexes I, II, III and V, as detected by immunoblotting (Fig. 5D).

We next asked whether, independently of differences in mitochondria numbers, the lower levels of OXPHOS protein complexes in disease [seen both in the nascent proteomes of whole hippocampi, neurons and astrocytes (Figs 4A and 5B) and in immunoblots (Fig. 5D)] are reflected in a functional effect on mitochondrial respiration. Likewise, we asked if the quantitative changes induced by trazodone are reflected functionally. We performed mitochondrial stress testing Seahorse XF96 Analyzer (Agilent) on mitochondria isolated from whole hippocampi of tg37^+/−^ control (NBH), prion-diseased and prion + trazodone mice at 10 wpi, using equal numbers from each condition. Mitochondrial respiration, measured as OCR, was recorded at baseline and in response to different stressors. Basal mitochondrial respiration was not significantly different between controls (81.1 pmol/min/ μg) and prion-diseased samples (68.2 pmol/min/ μg; *P* = 0.47), but trazodone treatment resulted in a significantly higher OCR (103.1 pmol/min/ μg; *P =* 0.0342) (Fig. 5E). No differences in respiration were observed between groups after injection of ADP or oligomycin, consistent with true OXPHOS impairment (Supplementary Fig. 4C). Injection of FCCP, an uncoupler that depolarizes mitochondria and maximizes OCR, resulted in significantly lower OCR in prion mitochondria than in controls (64.1 versus 117.2 pmol/min/ μg, *P* = 0.0274), which was restored to control levels by trazodone treatment (118 pmol/min/ μg, *P* = 0.0257) (Fig. 5E). Mitochondria from prion-diseased brains failed to increase their respiratory rate at all with FCCP, consistent with the reduction in OXPHOS proteins rendering ATP production maximal at baseline, with no spare capacity. When exposed to rotenone and antimycin A, which inhibit complexes I and III of the electron transport chain, OCR levels of all groups were drastically lowered below basal respiration with no differences between groups, as expected (Fig. 5E). Together, these data show that prion disease affects mitochondrial biology through reduction in net numbers and protein levels, as well as functionally, with reduced respiration—independent of number. Trazodone treatment rescues these defects, restoring mitochondrial capacity to sustain neurons and likely other cells in the hippocampus with crucial ATP production.

**Figure 6:**
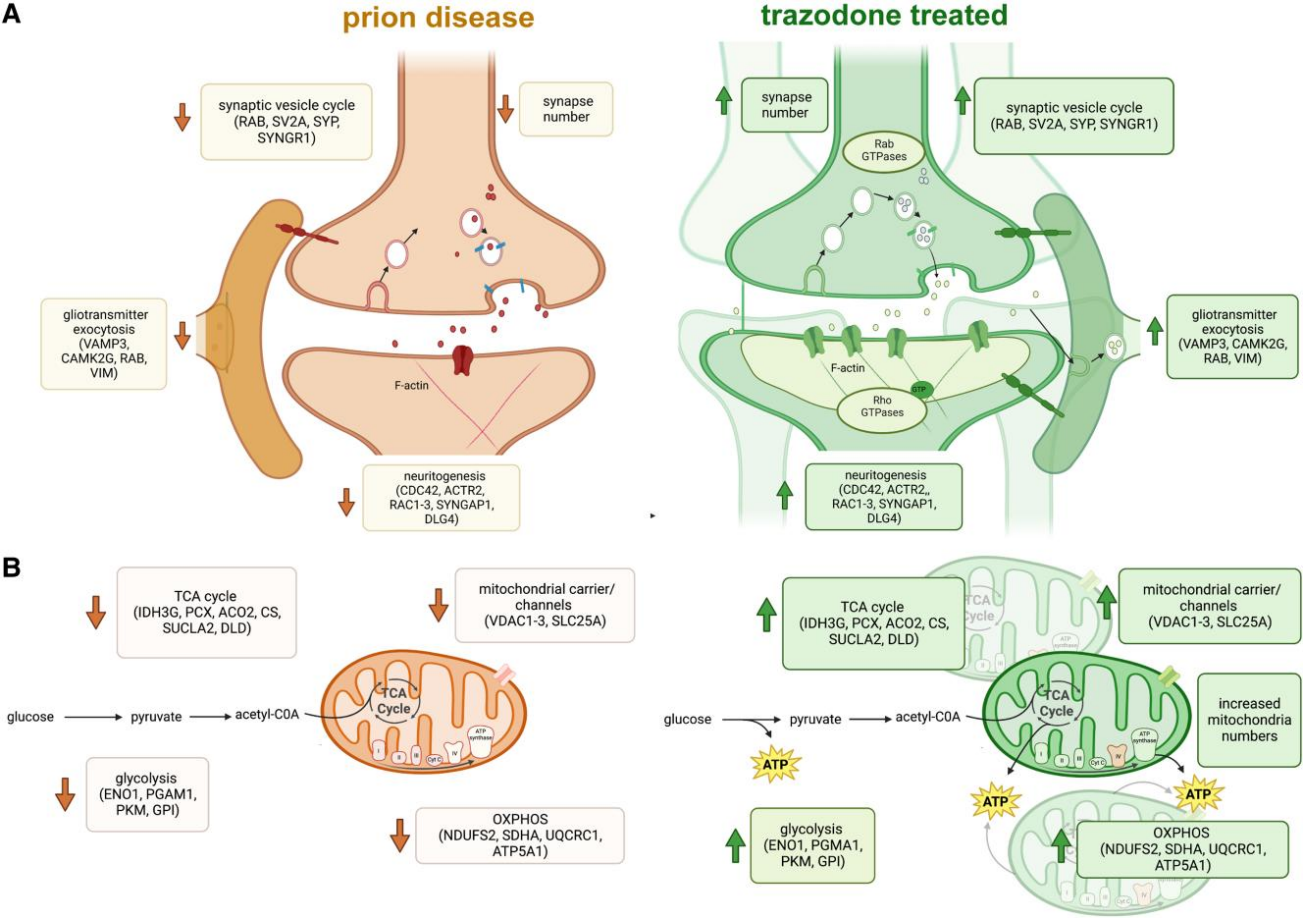
**Trazodone treatment in prion disease.** Schematic summarizing pathway and major proteins downregulated in the nascent proteome during prion disease and recovery after trazodone treatment for (**A**) synapses and (**B**) mitochondria.

The nascent proteome changes at the synapse and in mitochondria and their reversal by trazodone are in Fig. 6.

## Discussion

Repression of protein synthesis rates in neurodegenerative disease is increasingly accepted to be a contributing factor to impaired memory formation, synapse loss and neuronal demise across the spectrum of these disorders. Several pathways contribute to the failure of protein homeostasis (proteostasis), including those controlling both the initiation^58^ and elongation^59^ phases of translation, ribosomal function^60-63^ and protein degradation.^64^ Dysregulation of the control of the initiation phase of translation, in particular, via the PERK branch of the UPR, occurs across the spectrum of human neurodegenerative disease.^10^ In mouse models of neurodegenerative disorders, from Alzheimer’s, Parkinson’s, tauopathies, ALS and prion disease, overactivation of this pathway is associated with pathology and clinical signs. Genetic or pharmacological reversal restores global protein synthesis rates and is markedly neuroprotective despite high levels of ‘toxic’ misfolded proteins, highlighting the importance of helping restore proteostasis to mitigate the effects of proteotoxicity.^11,12,14-25,34,53^ Restoring translation at the level of elongation is also protective in Alzheimer’s mouse models,^35-37^ further speaking to the importance to neuronal survival of maintaining a healthy proteome. Yet the precise nature and identity of these dysregulated proteins has only recently begun to be explored^42-44^ and is an important focus for an increased understanding of the subcellular mechanisms and processes that lead to neuronal dysfunction and death.

In this study, we wanted to define the altered nascent proteome during disease—prior to overt neurodegeneration—and its response to treatment. Our hypothesis was that if UPR activation, known to be associated with synaptic failure and neuronal demise, leads to dysregulation of a specific set of proteins, then UPR-targeted treatments should, at least in part, reverse this. We were interested in both the neuronal and astrocytic specific proteomes as both cell types are critically involved in synapse formation and function, and modulating UPR activation in each is equally neuroprotective.^21,24^ We found proteins involved in two major sets of biological processes and subcellular structures to be dominantly affected by the disease processes: those involving the synapse and the mitochondria.

Synaptic dysfunction is the main cause of memory-associated cognitive deficits in neurodegenerative disease and is a prequel to neuronal demise.^65,66^ Our data show that during prion disease, when synapse loss is established but neuronal loss is imminent—10 wpi in tg37^+/−^ mice and 18 wpi in NCAT mice—that levels of predominantly neuronal synaptic proteins, both presynaptic (including SYN2, STX1B, SYNGR1, SYP) and postsynaptic (including SYNGAP1, DLG4, SLC6A11) are lowered by disease but restored by trazodone treatment (Fig. 3 and Supplementary Table 2). Concurrently, we observed an increase in levels of RAC1 and CDC42, Rho GTPases known for their role in F-actin assembly and disassembly important in bouton and dendritic spine formation^67,68^ by trazodone (Fig. 3 and Supplementary Table 2), suggesting that synaptic remodelling is also restored. Presynaptic membranes are also affected during disease with reduced levels of small Rab GTPases, which play a key role in the loading of neurotransmitters into synaptic vesicles,^69,70^ again reversed by trazodone treatment. Trazodone also reduced apoptotic signalling pathways upregulated in neurons during disease (Fig. 4 and Supplementary Table 4). Astrocytes are also key for synapse maintenance. They closely interact with neurons at both the pre- and postsynapse, in a structure known as the tripartite synapse.^71^ The tripartite synapse is critical during development to establish neuronal connectivity and in adult brains it integrates synaptic activity, contributes to the formation and elimination of synapses and releases gliotransmitters such as ATP, glutamate and serine in a Ca^2+^-dependent manner.^71^ Changes in gliotransmitter release are known to impact neuronal function and contribute non-cell autonomously to neurodegenerative disease.^72^ Indeed, prion disease produces a UPR-reactive astrocytic state that leads to a non-cell autonomous neuronal degeneration via an altered astrocytic secretome that is reversed by UPR inhibition, with recovery of synaptic and neuronal numbers and extended survival.^24^ Our BONCAT analysis of trazodone-treated astrocytes showed a restoration of key proteins involved in SNARE signalling (RAB2A, NSF, SYT3, VAMP3, ERC2), key for astrocytic exocytosis, supporting the hypothesis that trazodone rescues astrocyte neurotrophic support. Also restored are integrin signalling (ARF5, ARHGDIA, PFN2), important for tripartite synapse function (Fig. 4 and Supplementary Table 2). These data thus provide a detailed mechanistic underpinning at multiple levels of synaptic function and structural plasticity in both neurons and astrocytes for the functional recovery in memory we and others have previously shown with trazodone in prion-diseased^22^ and tauopathy rTg4510 mice,^22,34^ as well as protection from neurodegeneration. The protection from synapse loss seen in SEM images (Fig. 5A) by trazodone treatment is also explained by these changes in the proteome.

Also essential for both synaptic and neuronal function are healthy mitochondria. Mitochondrial dynamics, mobility and turnover are perturbed, and TCA cycle proteins and OXPHOS complexes are downregulated in prion and other neurodegenerative diseases in humans and mouse models.^73-81^ Mitochondria are commonly found at nerve terminals where they help maintain neurotransmission at the presynapse through Ca^2+^ efflux and the production of ATP.^82,83^ Both GTPase activity and increased protein synthesis rates in neurons require ATP and GTP, synthesized by mitochondria. The importance of glycolysis and mitochondrial metabolism for neurons and specifically for synapse function is well established.^73-77,82-84^ In a similar fashion, dendritic mitochondria are also essential at endoplasmic reticulum-mitochondrial contact sites, which regulate ATP and Ca^2+^ dynamics for the morphogenesis and plasticity of synaptic spines.^82,83^ Our BONCAT analyses and validation experiments reveal that trazodone treatment increases mitochondrial activity by increasing the expression of different proteins related to glycolysis (PKM, GPI, TPI1), TCA cycle enzymes (PCX, CS, DLAT, LDHA/B) as well as OXPHOS complexes (NDUFS1, SDHA, UQCRC2, MT-CO2, CYC1, ATP5J2) that are downregulated in prion disease (Fig. 3 and Supplementary Table 2), as also seen in the hamster tg7 prion model, which shows downregulation of OXPHOS complex I and V, as well as proteins involved in the TCA cycle.^78^ Consistent with this, trazodone protects from mitochondrial loss with restoration of reduced numbers to wild-type levels both within neurons and within dendrites (Fig. 5B and C). Critically, these changes in protein levels are reflected in mitochondrial functional output, with lower maximal respiratory capacity in mitochondria from diseased brains compared to controls (as also seen in tg7 prion hamsters^78^), which trazodone was able to reverse completely, restoring normal mitochondrial respiratory function as well as numbers (Fig. 5E).

Astrocytes are also an important hub for metabolism in the brain, providing many key metabolites to support synaptic and neuronal function, including ATP.^79^ Trazodone restored astrocytic mitochondrial proteins including those of TCA cycle and glycolysis (PKM, TPI1, IDH3G, SUCLG1) and oxidative phosphorylation (UQCRC1, MT-CO2, ATP5B, CYC1). This restoration of key metabolic processes in astrocytes likely aids in the synaptic protection discussed above. Interestingly, mitochondrial dysfunction, ultimately leading to a lowering in ATP production, has been described throughout the spectrum of neurodegenerative diseases,^75-78,80,81,85,86^ including Alzheimer’s,^43,44,75,76,81,85^ Parkinson’s,^87^ tauopathies^42,86^ and ALS.^77^

The proteome in various neurodegenerative diseases has been previously examined, including in prion disease—notably in human brains—where downregulation of pathways associated with Parkinson’s and Alzheimer’s disease, lysosomes and OXPHOS are reported.^88^ Mouse prion bulk proteomic data from whole brains showed changes in calcium signalling^40^ and neuroinflammation^41^ in terminal disease, but these studies do not address acute translational changes, which requires analysis of the nascent proteome, nor are they cell-specific. More recently and relevant to our study, the *de novo* nascent proteome has been examined using NCAT in mouse models of frontotemporal dementia (K3 FTD)^42^ and APP/PS1 model of Alzheimer’s disease.^43,44^ In K3 mice, AHA labelling of the whole brain proteome over 16 h in 5-month-old mice showed, similar to our findings, a large reduction in neuronal protein synthesis rates measured by FUNCAT.^42^ Proteomics analysis showed reduced differentially expressed proteins related to OXPHOS complexes, the TCA cycle, microtubules and actin cytoskeleton, synaptic proteins and ribosomal proteins, confirmed in rTg4510 mice, again similar to our findings. Hippocampal slices from APP/PS1 mice, examined by AHA labelling over 5 h combined with SILAC (BONLAC), again revealed changes in synapses, proteasome, lysosome, OXPHOS and ribosomal proteins.^43^ In another study in APP/PS1 mice, the AHA-labelled hippocampal proteome showed similar changes.^44^ Importantly, independent of neurodegenerative disease model used, anatomical area analysed and approach used to label the nascent proteome, there is a consistent and comprehensive overlap of proteins and cellular functions dysregulated across prion, tauopathy and Alzheimer’s disease models, stressing the mechanistic commonality of fundamental neurodegenerative processes across these disorders. However, none of the studies in Alzheimer’s disease and tauopathy models examined the response of the nascent proteome to therapies directed at restoring translation. Here, we have gained a deeper understanding of the proteins dysregulated by changing protein synthesis rates and their subsequent rescue with trazodone in both neurons and astrocytes in prion disease, supporting trazodone’s neuroprotective effects being due to synergistic protection in multiple cell types in the brain.

In conclusion, these data bring new insights into how translational repression during disease is so devastating to synaptic function and neuronal survival, via the chronic depletion of essential synaptic and mitochondrial proteins and ATP, resulting in loss of synapses, mitochondria and ultimately, neurons. They also bring new understanding of the specific mechanisms by which trazodone mediates neuroprotection: through restoration of these key proteins essential for neural function and resilience. Given the marked commonalities we found with the dysregulated proteomes in Alzheimer’s disease and tauopathy models, and the known underpinning of UPR dysregulation across these disorders, our results on treatment with trazodone have wide implications for therapy of neurodegenerative disorders.

## Supplementary Material

awad313_Supplementary_Data

## Data Availability

Processed data are available in Supplementary Tables 2–4. Raw data are available via the ProteomeXchange consortium with identifier PXD042577.
