# Supplementary material for: Trazodone rescues dysregulated synaptic and mitochondrial nascent proteomes in prion neurodegeneration
Source: Brain. 2023 Sep 13;147(2):649–64. doi: 10.1093/brain/awad313 (PMC10834243; doi:10.1093/brain/awad313)
Supplement: awad313_Supplementary_Data [file awad313_supplementary_data.zip › brain-2023-01047-File008.pdf]

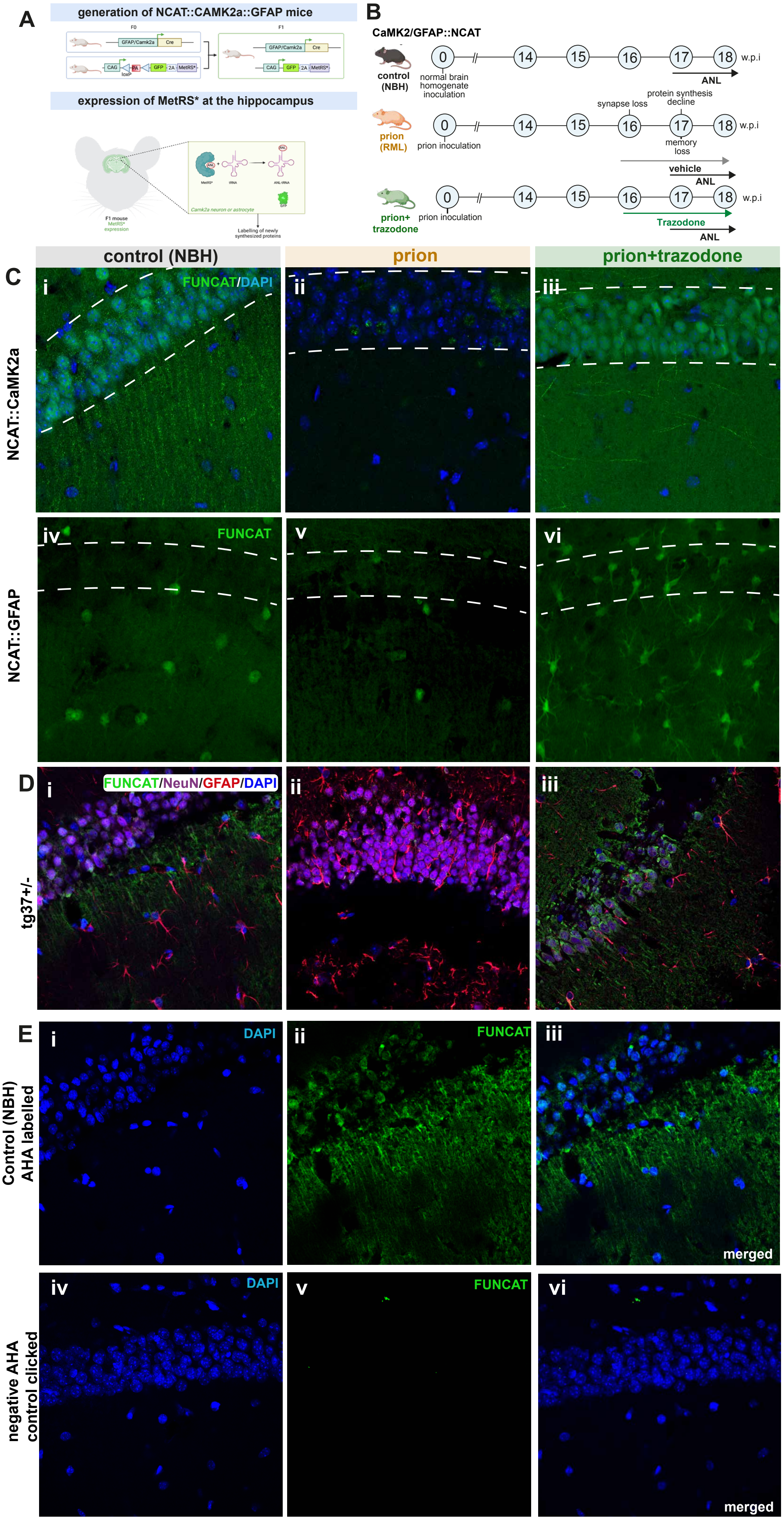

Supplementary figure 1. FUNCAT analysis. (**A**) Schematic of NCAT::GFAP/CaMK2a transgenic mice. (**B**) Schematic showing progression of prion disease, ANL labelling and trazodone treatment in NCAT::CaMK2a and NCAT::GFAP mice. (**C**) Representative images of FUNCAT analysis of the CA1 region from NCAT::CaMK2a (**i-iii**) and NCAT::GFAP (**iv-vi**) mice. (**D**) Representative FUNCAT/NeuN/GFAP/DAPI images from CA1 of AHA labelled control, prion and prion+trazodone mice. (**E**) Representative FUNCAT images from CA1 of AHA labelled control (**i-iii**) and negative AHA clicked animal (**iv-vi**) showing no FUNCAT signal in negative controls.

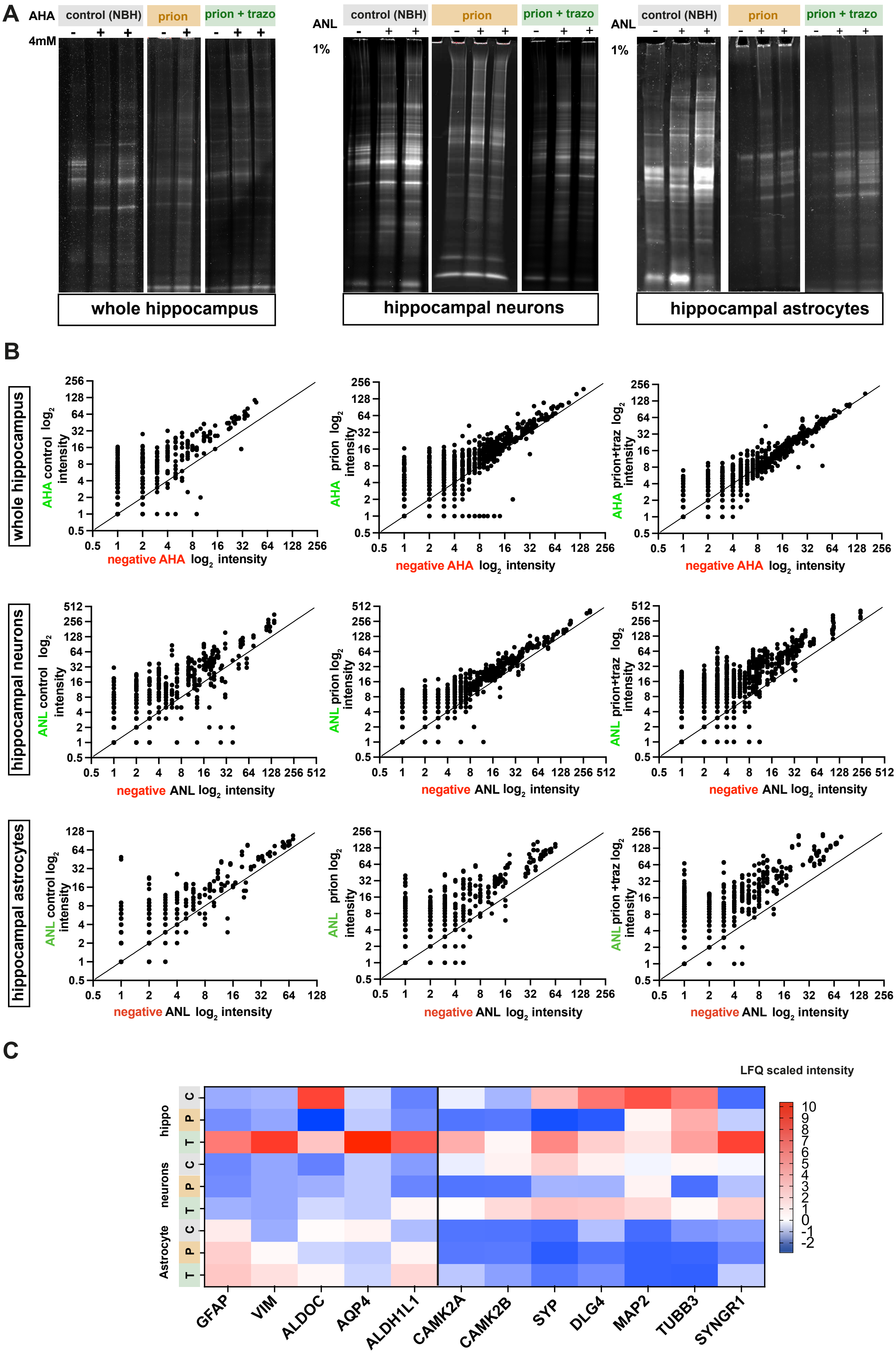

Supplementary figure 2. BONCAT elutions. (A) Representative elutions of the whole hippocampal, neuronal and astrocytic nascent translatomes, compared to negative controls. (**B**) ANL log2 intensity of detected proteins compared to negative AHA/ANL controls. (**C**) Heatmap of cell-specific LFQ scaled intensities markers identified on hippocampal, neuronal and astrocytic nascent proteomes. (C): control (NBH), P: prion and T: prion+trazodone.

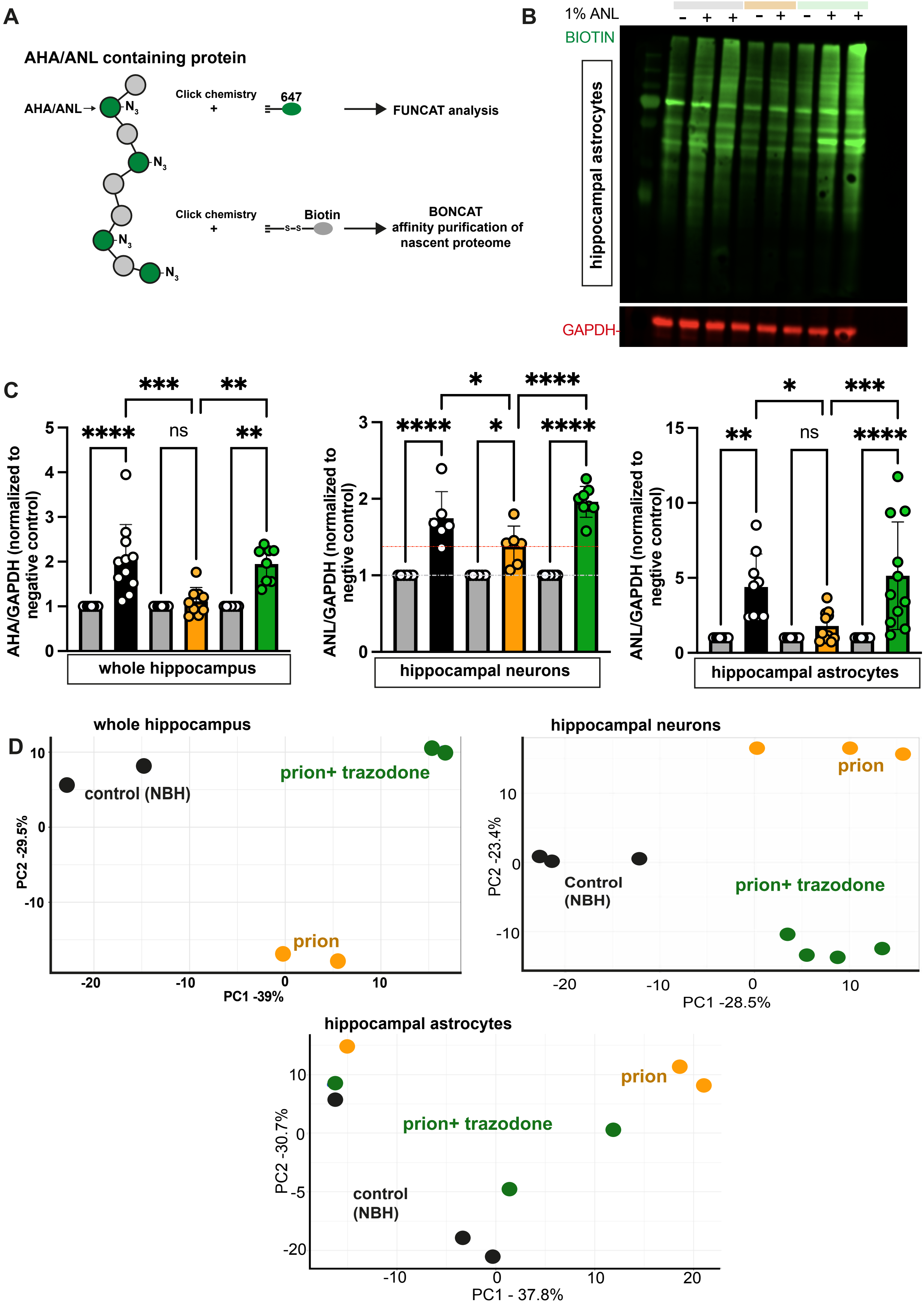

Supplementary figure 3. Hippocampal nascent translatome. (A) Schematic of click chemistry performed on AHA/ANL containing proteins analysed through either FUNCAT or BONCAT. (**B**) Immunoblot illustrating NCAT::GFAP ANL incorporation in control (NBH) (black line), prion (orange line) and prion + trazodone (green line) mice. (**C**) Immunoblot quantifications of AHA and ANL incorporations for whole, neuronal and astrocytic hippocampus nascent proteomes, normalized to negative control. (**D**) Principal component analysis plots of whole, neuronal and astrocytic hippocampus from all nascent proteomes. *p* values: * < 0.05, ** <0.001, *** <0.0001,**** <0.00001.

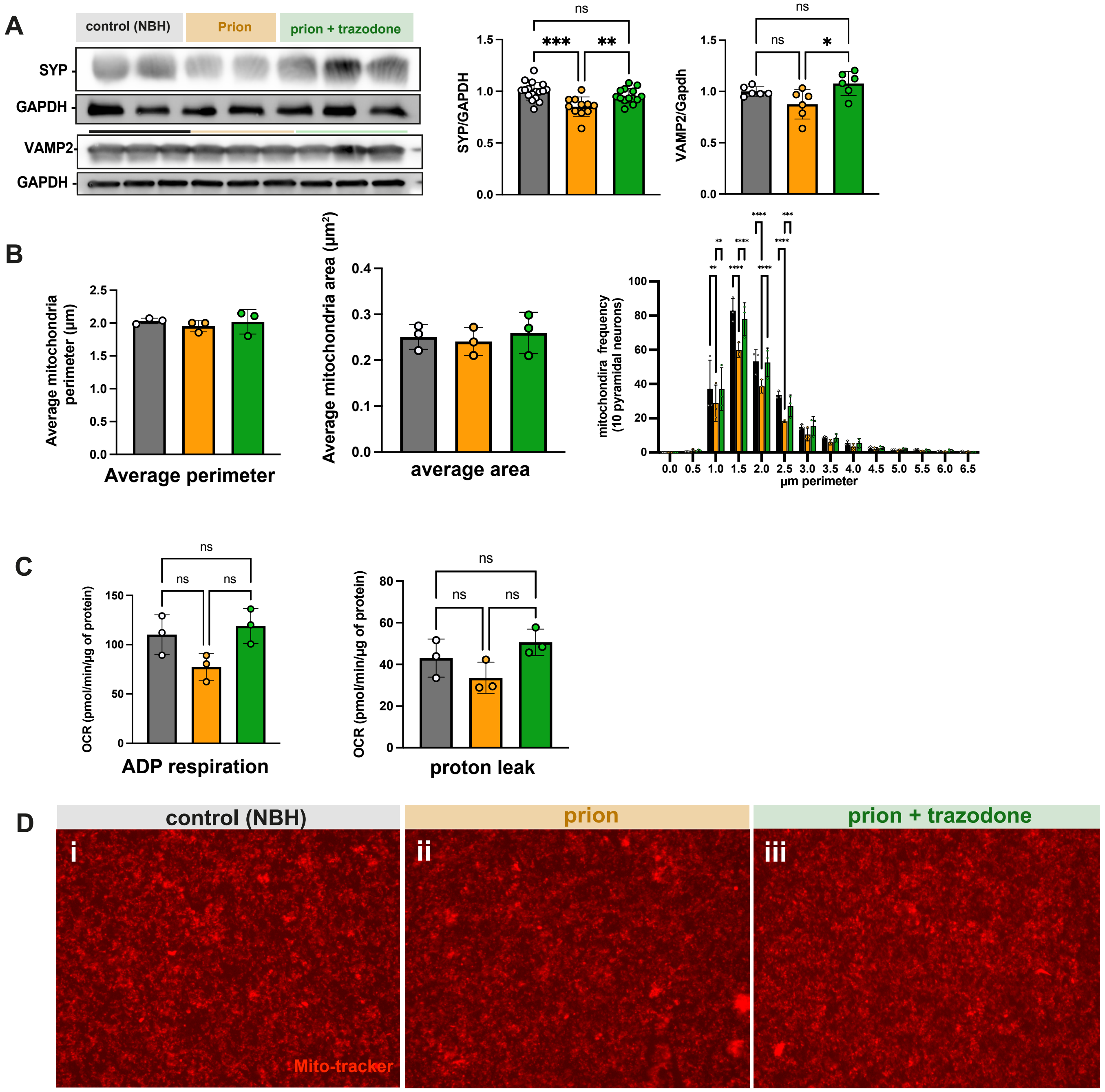

Supplementary figure 4. Trazodone restores synaptic proteins and mitochondria in prion disease. (A) Immunoblots and quantification of synaptic proteins (SYP and VAMP2) in 10w.p.i $tg37^{+/-}$ control, prion and prion+trazodone mice. (**B**) Average mitochondrial perimeter, area and frequency perimeter distribution in CA1 pyramidal neurons. (**C**) OCR from control (NBH), prion and prion+trazodone after ADP injection (left) and after oligomycin injection (right). (**D**) Images of isolated mitochondria from (**i**) control, (**ii**) prion and (**iii**) prion+trazodone $tg37^{+/-}$ mice using mitotracker dye. *p* values: * < 0.05, ** <0.001, *** <0.001
