# Supplementary material for: Trazodone rescues dysregulated synaptic and mitochondrial nascent proteomes in prion neurodegeneration
Source: Brain. 2023 Sep 13;147(2):649–64. doi: 10.1093/brain/awad313 (PMC10834243; doi:10.1093/brain/awad313)
Supplement: awad313_Supplementary_Data [file awad313_supplementary_data.zip › brain-2023-01047-File013.pdf]

Figure 5D - OXPHOS cocktail for 5 electron transport chain complexes

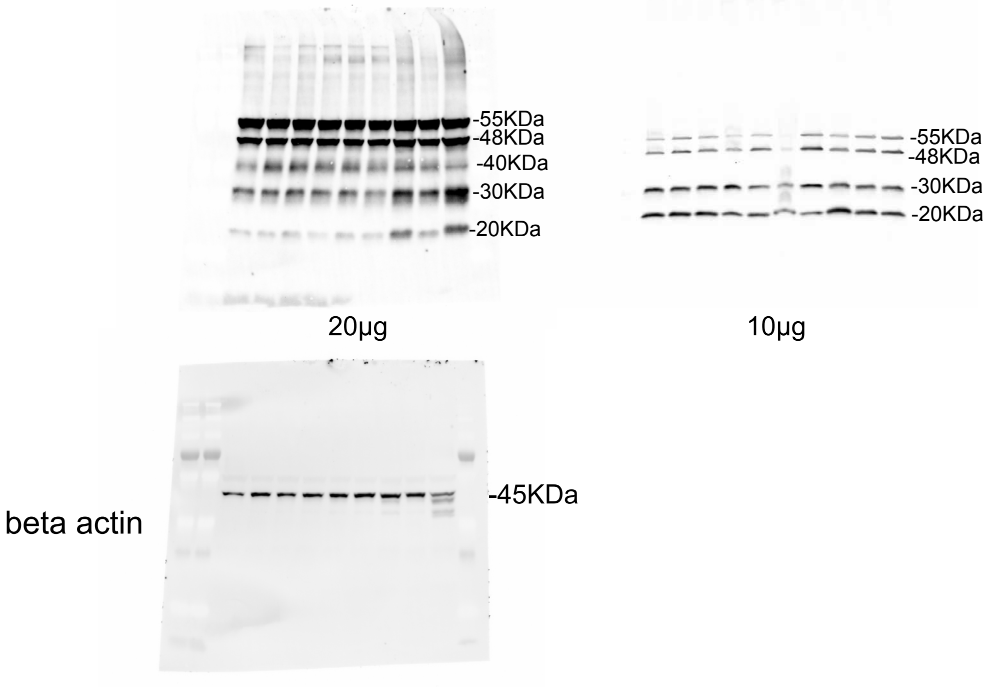

Supplementary Figure 2A - SyproRuby protein stains

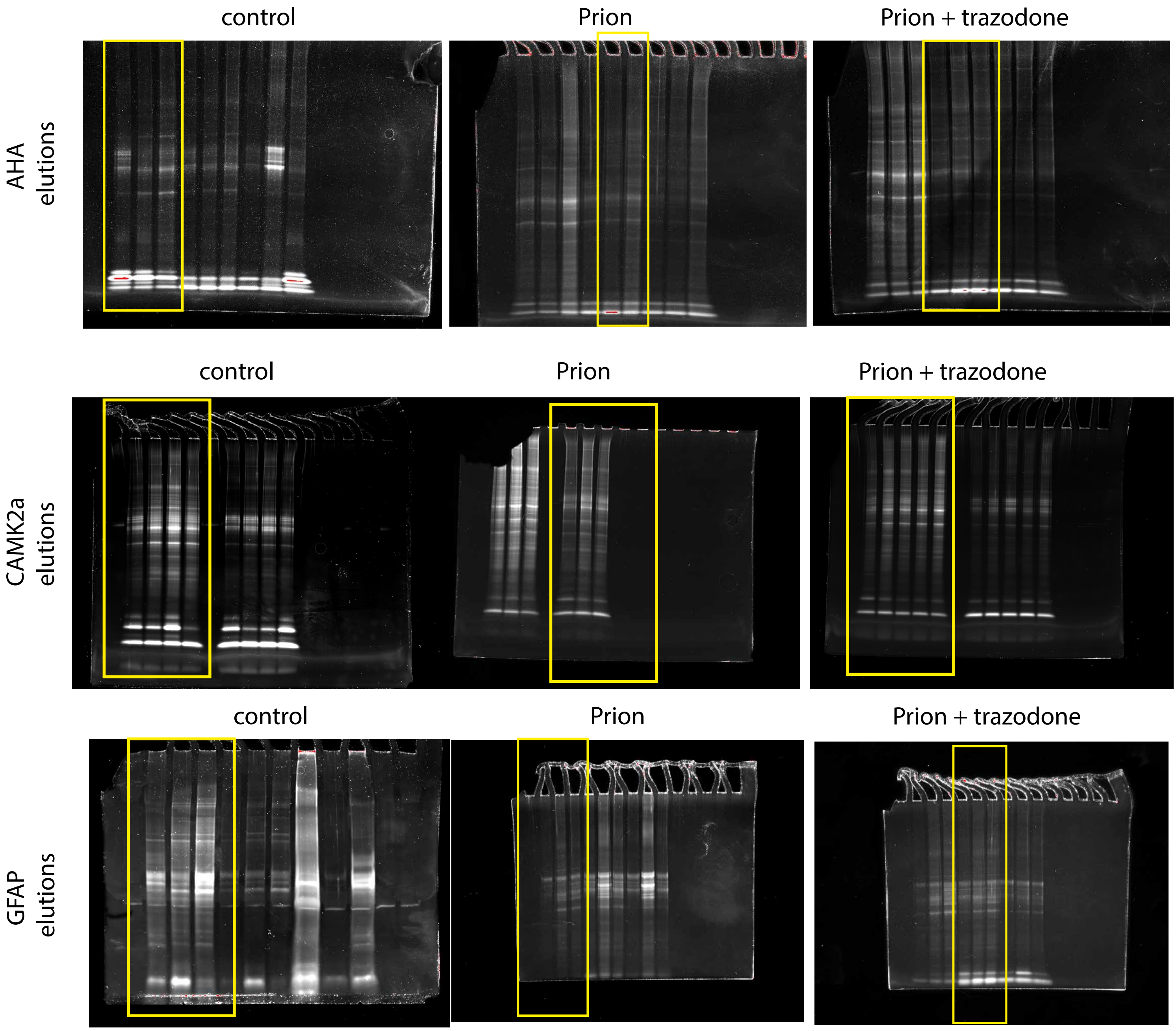

Supplementary Figure 3B - GFAP::NCAT ANL incorporation

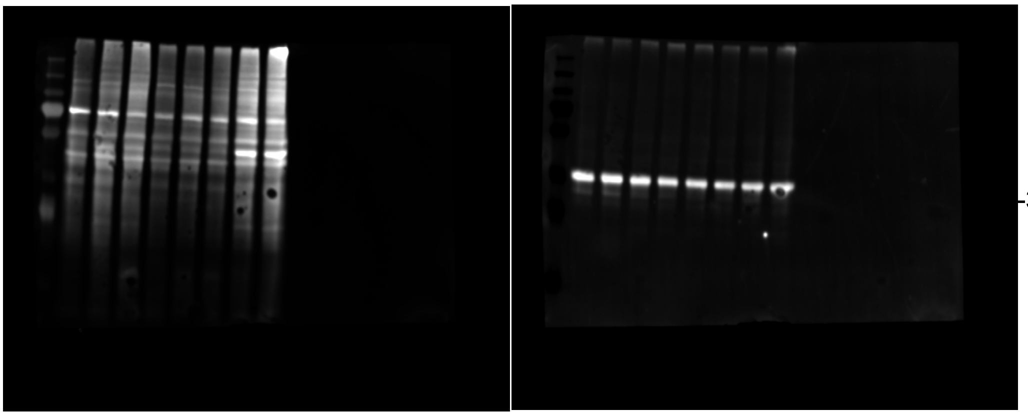

-37KDa

biotin GAPDH

Supplementary Figure 4A - SYP and VAMP2 immunoblots

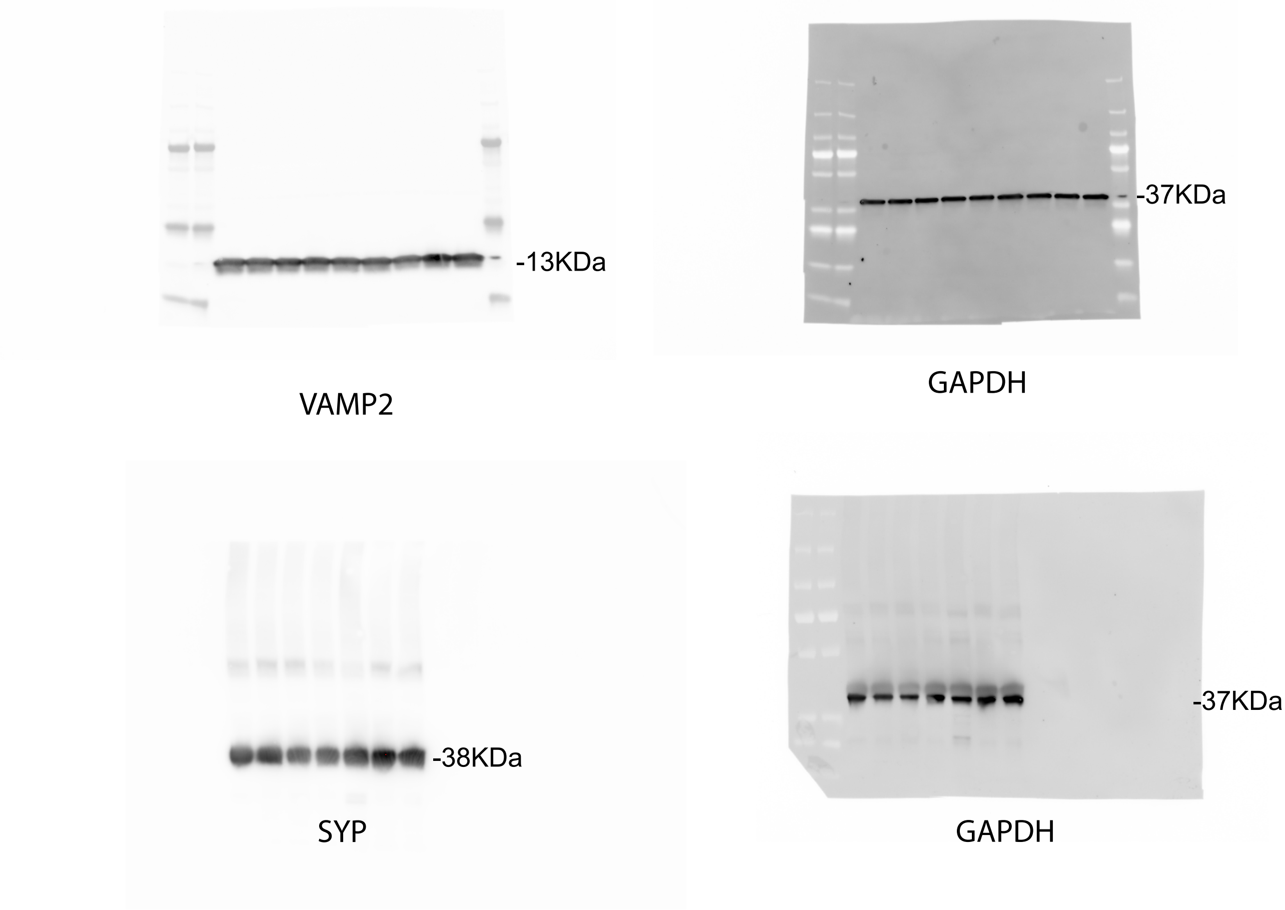

-13KDa -37KDa

VAMP2 GAPDH

-38KDa -37KDa

SYP GAPDH
